# Supplementary material for: Assessing the age specificity of infection fatality rates for COVID-19: systematic review, meta-analysis, and public policy implications
Source: Eur J Epidemiol. 2020 Dec 8;35(12):1123–38. doi: 10.1007/s10654-020-00698-1 (PMC7721859; doi:10.1007/s10654-020-00698-1)
Supplement: Supplementary file 1 — Supplementary file1 (PDF 669 kb) [file 10654_2020_698_MOESM1_ESM.pdf]

Assessing the Age Specificity of Infection Fatality Rates for COVID-19: Systematic Review, Meta-Analysis, and Public Policy Implications

Supplementary Appendix

Andrew T. Levin, William P. Hanage, Nana Owusu-Boaitey,
Kensington B. Cochran, Seamus P. Walsh, and Gideon Meyerowitz-Katz

23 November 2020

Corresponding Author:

Gideon Meyerowitz-Katz
Research, Monitoring & Surveillance Coordinator
Western Sydney Local Health District
PO Box 792
Seven Hills, NSW 2147 Australia
Phone: +61-2-9881-8878
Email: gideon.meyerowitzkatz@health.nsw.gov.au

List of Supplementary Appendices

A: PRISMA Checklist

B: Meta-analysis search procedure

C: Seroprevalence adjustments for test characteristics

D: Countries with comprehensive tracing programs

E: Prevalence vs reported cases in Iceland

F: Age-specific fatality data and source information

G: Metaregression methodology

H: List of excluded studies

I: Seroprevalence Rates for Studies Included in Meta-Analysis

J: Assessment of risk of bias for included studies

K: Assessment of publication bias

L: Stability of metaregression results across age categories

M: Out-of-sample analysis of metaregression results

N: U.S. scenario analysis

O: Infection fatality rate for seasonal influenza

P: Comparison of confirmed fatalities and excess mortality

Q: Comparison of age-specific IFRs

R: Comparison of age-specific CFR vs. IFR

S: Comorbidities and demographic factors

T: Metaregression Predictions for Manaus, Brazil

Supplementary Appendix A: PRISMA Checklist[1]

Section/topic	#	Checklist item	Page #
**TITLE**			
Title	1	Identify the report as a systematic review, meta-analysis, or both.	Title Page
**ABSTRACT**			
Structured summary	2	Provide a structured summary including, as applicable: background; objectives; data sources; study eligibility criteria, participants, and interventions; study appraisal and synthesis methods; results; limitations; conclusions and implications of key findings; systematic review registration number.	Title Page
**INTRODUCTION**			
Rationale	3	Describe the rationale for the review in the context of what is already known.	1-2
Objectives	4	Provide an explicit statement of questions being addressed with reference to participants, interventions, comparisons, outcomes, and study design (PICOS).	1-2
**METHODS**			
Protocol and registration	5	Indicate if a review protocol exists, if and where it can be accessed (e.g., Web address), and, if available, provide registration information including registration number.	NA
Eligibility criteria	6	Specify study characteristics (e.g., PICOS, length of follow-up) and report characteristics (e.g., years considered, language, publication status) used as criteria for eligibility, giving rationale.	2
Information sources	7	Describe all information sources (e.g., databases with dates of coverage, contact with study authors to identify additional studies) in the search and date last searched.	2, App.B
Search	8	Present full electronic search strategy for at least one database, including any limits used, such that it could be repeated.	App. B
Study selection	9	State the process for selecting studies (i.e., screening, eligibility, included in systematic review, included in the meta-analysis).	2-5, App. D
Data collection process	10	Describe method of data extraction from reports (e.g., piloted forms, independently, in duplicate) and any processes for obtaining and confirming data from investigators.	2, App. F, H
Data items	11	List and define all variables for which data were sought (e.g., PICOS, funding sources) and any assumptions and simplifications made.	2-5
Risk of bias in individual studies	12	Describe methods used for assessing risk of bias of individual studies and how this information is to be used in any data synthesis.	2-5
Summary measures	13	State principal summary measures.	1-5
Synthesis of results	14	Describe the methods of handling data and combining results of studies, if done, including measures of consistency.	5, App. G
Risk of bias across studies	15	Specify any assessment of risk of bias that may affect the cumulative evidence (e.g., publication bias, selective reporting within studies).	5, App. G
Additional analyses	16	Describe methods of additional analyses (e.g., sensitivity or subgroup analyses, meta-regression), if done, indicating which were pre-specified.	5, App. G

**RESULTS**			
Study selection	17	Give numbers of studies screened, assessed for eligibility, and included in the review, with reasons for exclusions at each stage, ideally with a flow diagram.	6, 9-10, App. J
Study characteristics	18	For each study, present characteristics for which data were extracted (e.g., study size, PICOS, follow-up period) and provide the citations.	7-9, App. F, H
Risk of bias within studies	19	Present data on risk of bias of each study and, if available, any outcome level assessment (see item 12).	App. I
Results of individual studies	20	For all outcomes considered (benefits or harms), present, for each study: (a) simple summary data for each intervention group (b) effect estimates and confidence intervals, ideally with a forest plot.	14-16
Synthesis of results	21	Present results of each meta-analysis done, including confidence intervals and measures of consistency.	11-13
Risk of bias across studies	22	Present results of any assessment of risk of bias across studies (see Item 15).	App. K
Additional analysis	23	Give results of additional analyses, if done (e.g., sensitivity or subgroup analyses, meta-regression [see Item 16]).	App. L
**DISCUSSION**			
Summary of evidence	24	Summarize the main findings including the strength of evidence for each main outcome; consider their relevance to key groups.	9-10
Limitations	25	Discuss limitations at study and outcome level (e.g., risk of bias), and at review-level (e.g., incomplete retrieval of identified research, reporting bias).	18
Conclusions	26	Provide a general interpretation of the results in the context of other evidence, and implications for future research.	17-18
**FUNDING**			
Funding	27	Describe sources of funding for the systematic review and other support (e.g., supply of data); role of funders for the systematic review.	Title Page

Supplementary Appendix B: Meta-analysis search procedure

To perform the present meta-analysis, we collected published papers and preprints regarding the seroprevalence and/or infection fatality rate of COVID-19. To identify these studies, we systematically performed online searches in MedRxiv, Medline, PubMed, and Google Scholar using the criterion *((“infection fatality rate” or “IFR” or “seroprevalence” or “antibodies”) and (“COVID-19” or “SARS-Cov-2”))*. We also used a search tool created by the University of Zurich for searching EMBASE using the same search criterion.[2] We identified other studies listed in reports by government institutions such as the U.K. Parliament Office.[3] Finally, we confirmed the coverage of our search by referring to two recent meta-analysis studies of the overall IFR for COVID-19, a recent meta-analysis of the ratio of measured seroprevalence to reported cases, and the SeroTracker global dashboard of SARS-CoV-2 seroprevalence studies.[4-7] Our search encompassed studies that were publicly disseminated prior to 25 September 2020. For cases in which a study was identified by the aforementioned search but age-specific seroprevalence was not found, an expanded search was performed to obtain those details using additional keywords (e.g., the location of the study). Data was extracted from studies by three authors and verified prior to inclusion.

Supplementary Appendix C: Seroprevalence adjustments for test characteristics

Studies of COVID-19 seroprevalence have utilized a variety of distinct test procedures. Enzyme-linked immunosorbent assays (ELISA) proceed by tagging antibody-antigen interactions with a reporter protein. Chemiluminescent immunoassays (CLA) work similarly by tagging the antigen-antibody interaction with a fluorescent protein. Lateral Flow Assays (LFA), also known as rapid diagnostic tests (RDT), produce a colored band upon antigen-antibody interaction. Microneutralization tests (MNT), recombinant immunofluorescent assays (RIA), and plaque reduction neutralization tests (PRNT) can be performed in laboratories and provide extremely accurate assessments.

Recognizing that SARS-Cov-2 is both novel and hazardous, public regulatory agencies have issued "emergency use authorizations" (EUA) to facilitate the rapid deployment of test kits. Subsequent studies by independent laboratories have reassessed the characteristics of these test kits, in many cases finding markedly different results than those of the manufacturer. Such differences reflect (a) the extent to which test results may be affected by seemingly trivial differences in its implementation, and (b) the extent to which serological properties may vary across different segments of the population. For example, a significant challenge in producing accurate tests is to distinguish COVID-19 antibodies from those associated with other coronaviruses (including the common cold). Consequently, the assessment of test characteristics may vary with seemingly innocuous factors such as the season of the year in which the blood samples were collected.

The reliability of a seroprevalence test depends on its *sensitivity* (the likelihood that the test correctly detects the virus in an infected person) and *specificity* (the likelihood that the test has a negative result for a uninfected person) as well as the true prevalence of the disease. For example, in a population where the true prevalence is low, a test with sensitivity below 100% will yield a substantial fraction of false positive results.

Some seroprevalence studies have reported results for *raw prevalence*, that is, the ratio P / N, where P denotes the number of positive test results and N denotes the number of individuals who were tested, with confidence intervals that solely reflect sampling uncertainty (i.e., variations associated with drawing a finite sample of N observations). However, such estimates and confidence intervals may be misleading in the absence of adjustments that reflect the sensivity and specificity of the test method (except in the case of "gold standard" methods with 100% specificity and 100% sensitivity). Indeed, a recent systematic review and meta-analysis found very substantial divergences in sensitivity and specificity of COVID-19 serological tests.[8]

If the sensitivity and specificity of the test are assumed to be known with certainty, then the *test-adjusted prevalence* can be computed using the Gladen-Rogan formula[9] as follows:

$$adjusted\ prevalence\ =\ \frac{raw\ prevalence\ +\ specificity - 1}{sensitivity\ + specificity - 1}$$

Likewise, the test-adjusted confidence interval can be computed by multiplying the raw confidence interval by the factor 1/(*sensitivity* + *specificity* – 1).

Some recent seroprevalence studies have reported test-adjusted results using the Gladen-Rogan formula.[10-15] However, a number of other seroprevalence studies have only reported raw prevalence results.[12, 16-20] To facilitate the consistency of our meta-analysis, we follow the same approach of adjusting those raw prevalence results using the Gladen-Rogan formula, as reported in the table below.

Nonetheless, the sensitivity and specificity of COVID-19 antibody test kits should generally not be treated as parameters known with certainty. Indeed, the U.S. Food and Drug Administration reports 95% confidence intervals for each of these test properties in its information sheet for all EUA test kits.[21] In particular, following the approach of Gelman and Carpenter (2020), we use a Bayesian procedure to compute seroprevalence estimates and confidence intervals that incorporate uncertainty about the sensitivity and specificity of the test kit used in each of these seroprevalence studies.[22, 23] As shown in Table C1, the contrast between the Gladen-Rogan vs. Bayesian results are most striking for study observations obtained using a relatively small sample size in a context of low prevalence. In some cases, the Bayesian confidence interval indicates that the level of seroprevalence cannot be distinguished from zero, and hence those observations are excluded from our metaregression (see Appendix X).

Table C1: Raw vs. test-adjusted measures of seroprevalence

Location	Age Group	Sample (N)	Prevalence (%) Raw	Prevalence (%) Rogan-Gladen	Prevalence (%) Bayesian	95% CI Lower Bound Raw	95% CI Lower Bound Rogan-Gladen	95% CI Lower Bound Bayesian	95% CI Upper Bound Raw	95% CI Upper Bound Rogan-Gladen	95% CI Upper Bound Bayesian
Belgium[24]	0-24	1263	6.0	6.7	5.7	4.2	4.7	2.0	8.6	9.6	8.4
	25-44	1710	5.9	6.6	5.4	4.2	4.7	1.9	8.3	9.2	8.0
	45-64	1831	6.2	6.9	5.6	4.7	5.2	2.1	8.3	9.2	8.3
	65-74	878	4.1	4.6	3.6	2.3	2.6	0.7	7.2	8.0	6.1
	75-84	816	7.0	7.8	7.0	4.2	4.7	3.4	11.7	13.0	10.1
	85+	809	13.2	14.7	14.2	8.9	9.9	9.8	19.6	21.8	18.7
France[25]	0–39	2262	10.8	12.0	NA	9.6	10.6	NA	12.1	13.5	NA
	40–49	2897	11.5	12.7	NA	10.3	11.4	NA	12.6	14.0	NA
	50–59	3019	5.8	6.5	NA	5.0	5.5	NA	6.7	7.4	NA
	60–69	3272	4.1	4.5	NA	3.4	3.8	NA	4.7	5.3	NA
	70+	3175	3.1	3.4	NA	2.5	2.7	NA	3.7	4.1	NA
Hungary[17]	14-39	3353	0.5	0.1	0.2	0.3	0.0	0.0	0.9	0.5	0.6
	40-64	4735	0.7	0.3	0.3	0.4	0.0	0.0	1.0	0.6	0.7
	65+	2386	0.8	0.4	0.4	0.4	0.0	0.1	1.3	0.9	0.9
Ireland[26]	12–29	519	1·9	1·5	NA	0·7	0·3	NA	3·1	2·7	NA
	30–49	746	1·6	1·2	NA	0·7	0·3	NA	2·5	2·1	NA
	50–69	686	1·6	1·2	NA	0·7	0·3	NA	2·5	2·2	NA
Italy[16]	0-19	9460	2.2	1.8	1.8	1.7	1.3	1.2	2.8	2.4	2.2
	20-29	11302	2.1	1.7	1.7	1.7	1.3	1.1	2.4	2.0	2.1
	30-49	13816	2.4	2.0	2.0	2.1	1.7	1.4	2.8	2.4	2.4
	50-59	10639	3.1	2.7	2.7	2.7	2.3	2.1	3.5	3.1	3.2
	60-69	8324	2.6	2.2	2.2	2.1	1.7	1.6	2.9	2.5	2.7
	70+	11118	2.5	2.1	2.1	2.1	1.7	1.5	2.9	2.5	2.5
Portugal[18]	0-9	404	2.0	2.2	1.8	0.8	0.9	0.1	5.4	6.0	3.9
	10-19	377	2.1	2.4	1.8	0.8	0.9	0.1	5.5	6.1	4.2
	20-39	377	0.8	0.9	0.8	0.1	0.1	0.1	4.8	5.3	2.3
	40-59	479	2.3	2.6	2.0	0.9	1.0	0.2	5.9	6.6	4.2
	60+	664	2.4	2.7	2.0	1.1	1.2	0.2	4.9	5.4	4.0

Table C1: Raw vs. test-adjusted measures of seroprevalence *(contd.)*

Spain[20]	0-9	1590	2.8	2.4	2.4	1.5	1.1	1.5	5.2	4.8	3.4
	10-19	4937	3.3	2.9	2.9	2.4	2.0	2.3	4.5	4.1	3.5
	20-29	4808	3.8	3.4	3.4	2.8	2.4	2.7	5.2	4.8	4.1
	30–39	6445	3.5	3.1	3.0	2.6	2.2	2.4	4.5	4.1	3.6
	40–49	9670	4.0	3.6	3.6	3.1	2.8	3.0	4.9	4.5	4.1
	50–59	9652	4.1	3.7	3.7	3.2	2.8	3.1	5.1	4.7	4.2
	60–69	7564	3.8	3.4	3.4	2.9	2.5	2.8	4.9	4.5	3.9
	70+	7293	3.5	3.1	3.1	2.3	1.9	2.5	5.4	5.0	3.6
Indiana, USA[19]	0-39	1011	1.4	1.0	1.0	0.7	0.3	0.2	2.2	1.8	1.9
	40-59	1301	1.1	0.7	0.7	0.5	0.1	0.1	1.8	1.4	1.4
	60+	1206	0.7	0.3	0.4	0.3	0.0	0.0	1.3	0.9	1.1

Supplementary Appendix D: Countries with comprehensive tracing programs

Country	Cases *(thousands)*	Tests per confirmed case
New Zealand	1·1	1862
Australia	6·7	1054
South Korea	10·8	576
Lithuania	1·4	415
Iceland	1·8	321
Slovakia	1·4	194
Latvia	0·8	191
Austria	15·4	115
Slovenia	1·4	112
Czech Republic	7·6	104
Greece	2·6	95
Denmark	9·0	94
Estonia	1·7	70
Luxembourg	3·8	68
Israel	15·8	57
Norway	7·7	47
Poland	14·0	37
Hungary	2·8	36
Portugal	24·7	35
Belgium	49·9	32
Germany	159·1	31
Finland	4·9	31
Switzerland	29·3	27
Spain	215·2	27
Japan	14·1	25
Italy	203·6	19
Colombia	6·2	16
Canada	51·6	15
Ireland	20·3	13
Turkey	117·6	13
Chile	14·9	13
United Kingdom	167·2	10
Netherlands	38·8	8
United States	1039·9	8
Mexico	17·8	4
France	129·6	NA
Sweden	21·7	NA

Note: This table reports data for all OECD countries as of 30 April except Lithuania (28 April) and Poland (5 May); data on tests per confirmed case was not available for France and Sweden.[27] A national seroprevalence study of the Czech Republic found that infections exceeded confirmed cases by a factor of 5, suggesting that comprehensive tracing requires substantially more than 100 tests per confirmed case. By contrast, a seroprevalence study of Iceland indicates that its tracing program was effective in identifying a high proportion of SARS-CoV-2 infections.[28] For example, the Korea Center for Disease Control analyzed a total of 2995 serum samples collected for the National Health and Nutrition Examination Survey between 21 April and 13 August, and only one of those specimens tested positive for antibodies and neutralizing antibodies.[29] Similarly, a seroprevalence study of 1500 outpatients in Seoul found only one positive result.[30]

Supplementary Appendix E: Prevalence vs reported cases in Iceland

Age Group	Reported Cases	Estimated Infections	Confidence Interval		Ratio of Infections to Reported Cases	Confidence Interval	
			Lower	*Upper*		*Lower*	*Upper*
30-39	289	469	*469*	*703*	1·6	*1·6*	*2·4*
40-49	357	644	*473*	*859*	1·8	*1·3*	*2·4*
50-59	306	337	*211*	*547*	1·1	*0·7*	*1·8*
60-69	213	225	*188*	*375*	1·1	*0·9*	*1·8*
70-79	63	70	*63*	*304*	1·1	*1·0*	*4·8*
80+	25	26	*13*	*319*	1·0	*0·5*	*12·8*
All 30+	1253	1771	*1415*	*3109*	1·41	*1·13*	*2·48*

Sources: Cases are reported by Iceland Directorate of Health as of 14 June 2020, when Iceland had 1796 recovered cases, 10 fatalities, and 4 individuals in isolation (none hospitalized).[31] Estimated infections and 95% confidence intervals are taken from the prevalence study of Gudbjartsson et al. (2020), which conducted tests of a random sample of the general population on 16–31 March 2020.[32] As of 21 April 2020 (three weeks after the conclusion of that study), there were 1785 reported cases (98·6% of the total reported cases as of 14 June 2020).

Supplementary Appendix F: Age-specific fatality data and source information

F.1: Fatality data for European seroprevalence studies with representative samples

Location and source of fatality data	Study midpoint date in 2020	Fatality reporting date in 2020	Age group, years	Fatalities
Belgium[33]	April 23	May 16	0–24	1
			25–44	30
			45–64	409
			65–74	1061
			75–84	2144
			85+	5087
England[34]	July 1	July 29	0–17	11
			18–24	30
			25–34	131
			35–44	394
			45–54	1348
			55–64	3605
			65–74	7631
			75+	38629
France[35-38]	May 14	May 31	0–39	78
			40–49	167
			50–59	610
			60–69	1494
			70+	15050
Geneva, Switzerland[39]	April 26	June 1	0–19	0
			20–49	2
			50–64	16
			65+	268
Ireland[40]	July 4	August 2	15–44	19
			45–64	98
Italy[34]	July 16	August 13	0–19	4
			20–29	16
			30–49	369
			50–59	1186
			60–69	3433
			70+	29134
Netherlands[34]	April 9	May 7	0–49	40
			50–59	137
			60–69	454
			70–79	1539
			80+	2426
Portugal[34]	June 14	July 12	0–9	0
			10–19	0
			20–39	4
			40–59	75
			60+	1581
Spain[41]	May 25	July 15	0–9	5
			10–19	6
			20–29	35
			30–39	77
			40–49	295
			50–59	1023
			60–69	3049
			70+	24647

Notes: (1) The France seroprevalence study encompassed the regions of Grand Est, Ile de France, and Nouvelle Aquitaine. Total confirmed COVID-19 fatalities (including deaths in hospitals and in other medical and social institutions) were reported by region as of May 31.[35] The age composition of hospital fatalities for each region (published on June 3, May 28, and May 28, respectively) was used to compute total age-specific fatalities as of May 31.[36-38] Fatalities in other medical and social institutions were allocated to the 70+ age group. (2) Spain had 29137 laboratory-confirmed COVID-19 fatalities as of 15 July 2020, including 9909 deaths in nursing homes. Age-specific fatalities outside of nursing homes are reported by Pastor-Barriuso et al. (2020).[41] Abellán et al. (2020) estimated that 4% of Spanish nursing home residents were aged 65-69 while the remaining 96% were aged 70+.[42] We used those proportions to allocate the 9909 nursing home deaths to the corresponding age groups.

F.2: Fatality data for European and Canadian seroprevalence studies with convenience samples

Location and source of fatality data	Study midpoint date in 2020	Fatality reporting date in 2020	Age group, years	Fatalities
France[43, 44]	April 12	May 31	0–9	3
			10–19	3
			20–29	21
			30–39	84
			40–49	231
			50–59	860
			60–69	2204
			70–79	5650
			80+	19746
Ontario[45]	June 17	July 15	0–19	1
			20–59	122
			60+	2600
Sweden[46]	May 10	June 18	0–19	1
			20–49	63
			50–69	504
			70+	4485

Notes: France had a total of 28802 confirmed COVID-19 fatalities as of 31 May 2020, including 18475 deaths in hospitals and 10327 deaths in other medical and social institutions; France does not have real-time reporting of deaths in personal homes.[44] Age-specific COVID-19 fatalities are only reported for hospitals; therefore, age-specific COVID-19 fatalities for other medical and social institutions are allocated proportionally for ages 70–79 and 80+ based on the relative incidence of overall excess mortality during weeks 10 to 22 for those two age groups as reported by Eurostat (which collects that data daily from France Santé Publique).[43]

F.3 Fatality data for U.S. seroprevalence studies

Location and source of fatality data	Study midpoint date in 2020	Fatality reporting date in 2020	Age Group	Fatalities
Atlanta[47]	May 1	May 31	0–17	1
			18–49	20
			50–64	51
			65+	294
Connecticut[48]	April 30	May 28	0–19	2
			20–49	75
			50–59	157
			60+	3633
Indiana[49]	April 27	May 25	0–39	20
			40–59	148
			60+	1864
Louisiana[50]	April 5	May 6	0–18	1
			19–49	85
			50–59	126
			60+	1053
Miami[51]	April 8	May 6	0–18	0
			19–49	61
			50–59	169
			60+	1060
Minneapolis[52]	May 5	June 4	0–18	0
			19–49	18
			50–59	47
			60+	928
Missouri[53]	April 23	May 23	0–19	0
			20–49	18
			50–59	43
			60+	620
New York[54]	April 23	May 21	0–19	12
			20–39	482
			40–49	1026
			50–59	2764
			60+	24376
Philadelphia[55, 56]	April 19	May 17	0–18	1
			19–49	57
			50–59	323
			60+	2639
Salt Lake City[57]	May 22	June 19	0–44	4
			45–64	31
			65+	90
San Francisco[58]	April 25	May 25	0–18	0
			19–49	25
			50–59	66
			60+	333
Seattle[59]	March 27	April 26	0–19	0
			20–39	8
			40–59	69
			60+	700

Note: Some seroprevalence age brackets were adjusted (+/- 5 years) to match the age structure of that location's COVID-19 fatality report. For Pennsylvania and Utah, county-level data as of May 17 is available for total fatalities but not reported by age group; consequently, the statewide age distribution of fatalities was used to allocate county-level fatalities by age for each of those locations.

F.4 Fatality data for comprehensive tracing countries

Location and source of fatality data	Reporting date	Age group, years	Fatalities
Australia[60]	June 12	0–39	0
		40–59	3
		60–69	13
		70–79	31
		80+	55
Iceland[31]	June 14	0–29	0
		30–59	1
		60–69	2
		70–79	3
		80+	4
Korea[61]	July 11	0–29	0
		30–39	2
		40–49	3
		50–59	15
		60–69	41
		70–79	84
		80+	144
Lithuania[62]	June 21	0–39	0
		40–49	1
		50–59	3
		60–69	12
		70–79	23
		80+	37
New Zealand[63]	July 9	0–59	0
		60–69	3
		70–79	7
		80+	12

Note: Age-specific fatality data for Lithuania was published as of 01 June 2020, at which point there was a total of 70 reported fatalities; thus, the six subsequent fatalities through 22 June 2020 were assumed to have the same age distribution as the fatalities through 01 June 2020.

Supplementary Appendix G: Metaregression methodology

To analyze IFR by age, we use meta-regression with random effects, using the *meta regress* procedure in *Stata* v16.[64, 65] We used a random-effects procedures to allow for residual heterogeneity between studies and across age groups by assuming that these divergences are drawn from a Gaussian distribution. The procedure provides reasonable results even if the errors are not strictly normal but may be unsatisfactory if the sample includes large outliers or the distribution of groups is not unimodal. In analytical terms, this framework can be expressed as follows:

$$log(IFR_{ij}) = \alpha + \beta * age_{ij} + \epsilon_{ij} + u_{ij}$$

$$\text{where } u_{ij} \sim N(0, \tau^2) \text{ and } \epsilon_{ij} \sim N(0, \sigma_{ij}^2)$$

In this specification, IFR_{ij} is the estimated IFR in study *i* for age group *j*, age_{ij} denotes the median age of that group, ϵ_{ij} denotes the source of idiosyncratic variations for that particular location and age group, and u_{ij} denotes the random effects that characterize any systematic deviations in outcomes across locations and age groups. Under the maintained assumption that each idiosyncratic term ϵ_{ij} has a normal distribution, the idiosyncratic variance is $\sigma_{ij}^2 = ((U_{ij} - L_{ij})/3.96)^2$, where U_{ij} and L_{ij} denote the upper and lower bounds of the 95% confidence interval for that study-age group. The random effects u_{ij} are assumed to be drawn from a homogeneous distribution with zero mean and variance τ^2. The null hypothesis of $\tau^2 = 0$ characterizes the case in which there are no systematic deviations across studies or age groups. If that null hypothesis is rejected, then the estimated value of τ^2 encapsulates the magnitude of those systematic deviations.

Under our baseline specification, the infection fatality rate increases exponentially with age—a pattern that has been evident in prior studies of age-specific case fatality rates.[66, 67] Consequently, our meta-regression is specified in logarithmic terms, with the slope coefficient β encapsulating the impact of higher age on *log(IFR)*. Consequently, the null hypothesis that IFR is unrelated to age can be evaluated by testing whether the value of β is significantly different from zero. If that null hypothesis is rejected, then the estimated values of α and β characterize the estimated relationship between log(IFR) and age. Consequently, the predicted relationship between IFR and age can be expressed as follows:

$$IFR = e^{\alpha + \beta * age_{ij}}$$

The 95% confidence interval for this prediction can obtained using the delta method. In particular, let IFR_a denote the infection fatality rate for age a, and let σ_c denote the standard error of the meta-regression estimate of log(IFR_a). If IFR_a has a non-zero value, then the delta method indicates that its standard error equals σ_c / IFR_a , and this standard error is used to construct the confidence interval for IFR_a at each age a. Likewise, the prediction interval for log(IFR_a) is computed using a standard error of $\sigma_c + \tau$ that incorporates the systematic variation in the random effects across studies and age groups, and hence the corresponding prediction interval for IFR_a is computed using a standard error of $(\sigma_c + \tau)/IFR_a$.

In estimating this metaregression, we exclude observations for which the lower bound of the 95% confidence interval for seroprevalence of that particular age group equals zero, and hence the upper bound of that age-specific IFR is not well defined. Similarly, we exclude observations for which the lower bound of the 95% confidence interval for seroprevalence is less than the observed COVID-19 mortality rate for that age group, since such observations would imply an upper bound for the IFR that exceeds 100%. Finally, we exclude observations for which no COVID-19 fatalities were recorded for a given age group and hence the implied value of the infection fatality rate is at its lower bound of zero and the corresponding confidence interval cannot be precisely determined.

Supplementary Appendix H: List of excluded studies

H.1 Studies excluded due to absence of age-specific prevalence or fatality data

Location	Description
Alberta, Canada[68]	On July 30, Alberta's chief medical officer announced the results of a serology study of 9400 blood specimens collected for other purposes and indicated that "less than one percent" were positive. No further details were available as of September 17, 2020.
Ariano Irpino, Italy[69]	This seroprevalence study collected specimens in late May from 13444 individuals (about 75% of municipality residents) and found a raw prevalence of 4·83%. No age-specific results were reported.
Australia[70]	This study analyzed specimens from 2991 individuals undergoing elective surgery during May–June 2020 and found test-adjusted seroprevalence of 0·28% (CI: 0–0·71%). No age-specific seroprevalence results were reported. Although the sample of patients may not be fully representative of the Australian population, these results confirm that prevalence in Australia was indistinguishable from zero at the time of the study, as expected for a comprehensive tracing program (see Supplementary Appendix C).
Austria[71]	Statistik Austria conducted an experimental study in which specimens were collected from 269 individuals ages 16+. The test-adjusted seroprevalence was 4·71% (CI: 1·36–7·97%). No age-specific results were reported.
Bad Feilnbach, Germany[72]	This study collected specimens from a random sample of 2153 adults between 23 June and 4 July 2020 and found raw seroprevalence of 6·0%. No age-specific results were reported as of September 17.
Baton Rouge, Louisiana, USA[73]	This study analyzed specimens from a random sample of 2138 individuals between July 15–31 and found seroprevalence of 3·6%. No age-specific seroprevalence results were reported.
Blaine County, Idaho, USA[74]	This study collected specimens from 972 individuals on May 4-19 and found an IgG prevalence of 22·7% (CI: 20–25·5%). The authors concluded that "the small number of county deaths (*n*=5) makes estimating the infection fatality rate unreliable." No age-specific fatality data is publicly available for this county.
Bolinas, California, USA[75]	This study collected specimens from a random sample of 1312 confirmed residents of Bolinas on April 20-24. The test-adjusted seroprevalence was 0·16% (CI: 0·02–0·46%). No age-specific seroprevalence results were reported.
Bonn, Germany[76]	This study analyzed specimens from 4771 individuals participating in the Rhineland Study, an ongoing community-based prospective cohort study of Bonn residents ages 30 years and older. Specimens were collected between 24 April and 30 June 2020 and analyzed using a combination of ELISA, RIA, and PRNT methods. The ELISA assay found 46 positive results and indicated seroprevalence of 0·97% (CI 0·72–1·30%). By contrast, the PRNT assay found only 17 positive results and indicated a markedly lower seroprevalence of 0·36% (CI: 0·21–0·61%). No age-specific seroprevalence results were reported.
British Columbia, Canada[77]	This study analyzed 885 laboratory specimens from outpatient clinics for the period May 15-27 and found only four positive cases (0·6%). No age-specific seroprevalence was reported.
Burlington, Vermont, USA[78]	This study analyzed specimens from 454 primary care patients at a Level 1 medical center in Burlington on 25–28 June 2020 and found 10 positive results using a two-step serologic assay and found raw prevalence of 2·2 percent (CI: 0·8–3·6%). The median age of individuals with positive vs. negative results were nearly identical (51·9 vs. 51·4 years).
Caldari Ortona, Italy[79]	This study collected specimens from 640 residents on April 18-19 and found raw prevalence of 12%. No age-specific results were reported.
Chelsea, Massachusetts, USA[80]	This study collected specimens from 200 pedestrians and found a raw seroprevalence of 22·5% using an IgG LFA. No age-specific results were reported.

Connecticut,<br>USA[81]	This study analyzed specimens from a random sample of 505 adults residing in non-congregate settings. The sample design reflected the assumption of statewide prevalence of 10% (roughly similar to that of the neighboring state of New York) with the aim of obtaining prevalence estimates with precision of 2% at a confidence level of 90%. However, the study obtained a much lower estimated prevalence of 3·1% (95% CI: 1·1–5·1%). Consequently, the sample size proved to be insufficient to provide reliable age-specific results; the margin of error exceeds the estimated prevalence for all age groups reported in the study.
Czech<br>Republic[82]	The Czech Ministry of Health conducted a large-scale seroprevalence survey on April 23-May 1, collecting specimens from a random sample of 22316 residents and testing for IgG antibodies using the Wantai test kit. Only 107 positive cases were identified (raw prevalence = 0·4%), and hence the test-adjusted confidence intervals include the lower bound of zero prevalence. That result is consistent with the very low number of reported cases in the Czech Republic as of early May; for example, Prague had only 1,638 reported cases for a population of 1·3 million.
Denmark[83]	This study analyzed specimens from a random sample of 2427 individuals in early June and identified 34 positive cases, yielding a test-adjusted prevalence of 1·2% (CI: 0·7–1·7%). Age-specific estimates were not reported as of September 17.
Faroe Islands<br>Denmark[84]	This study analyzed specimens from a random sample of 1075 participants during late April and obtained 6 positive results; the test-adjusted prevalence was 0·7% (CI: 0·3–1·3%). No age-specific results were reported.
Finland[85]	Finland National Institute for Health and Welfare has been conducting an ongoing study of seroprevalence using random sampling of the population. Each specimen is initially screened for antibodies using a rapid test, and all specimens with positive screening results are analyzed using a microneutralization test (MNT) with confirmed specificity of 100%. As of August 8, this process screened 3155 specimens and obtained 8 positive MNT results (0.25%). No age-specific results were reported as of September 17.
Gangelt,<br>Germany[86]	This study analyzed specimens from a random sample of 919 participants from the municipality of Gangelt (population 12,597) on March 31 to April 6 and obtained a test-adjusted prevalence of 15·5% (CI: 12·3–19·0%). Official government reports indicate that Gangelt had 7 COVID-19 fatalities at the time of the study but the death toll rose to 12 by late June, indicating an overall IFR of about 0·6%, similar to the IFR for Geneva. Age-specific fatalities have not been reported for Gangelt.
Greece[87]	This study analyzed residual serum specimens from 6586 individuals collected during March and April and found 24 positive results. The test-adjusted prevalence was 0% (CI: 0–0·23%). Prevalence was reported for four age groups (0–29, 30–49, 50–69, and 70+); each of those confidence intervals included the lower limit of 0%.
Hibino,<br>Japan[88]	This study analyzed specimens from 615 employees of a Japanese company in Tokyo, including 350 individuals who were tested on two separate occasions. Among the 95 individuals with positive IgG results on the first occasion, there were 12 with negative IgG results in their second test. Among the 252 with an initial negative result, 54 had positive IgG results in their second test. No age-specific test results were reported.
Hideaway,<br>Texas USA[89]	This study analyzed specimens from 293 adults and found 7 positive results. Age-specific results were not reported.
Ischgl,<br>Austria[90]	This study analyzed specimens from 184 adults in Ischgl (an Austrian municipality of 1,604 residents) and obtained 85 positive results, i.e., prevalence of 46·2%. The study reported the fraction of positive results for specific age groups (4 out of 11 adults 55-64 years, 2 out of 8 adults 65-74 years, and 1 out of 2 adults ages 75+) but did not report test-adjusted estimates or confidence intervals by age group. Ischgl had only 2 reported COVID-19 fatalities as of July 1.
Israel[91]	Israel Health Ministry initiated a large-scale seroprevalence study in May. Subsequent media reports indicated that initial tests of 70000 Israelis indicated that prevalence varied significantly across regions and health organizations. No age-specific results had been released as of September 17.
Japanese Evacuees[92]	This study performed PCR tests on 565 Japanese citizens expatriated from Wuhan, China. There were eight positive tests, indicating a raw prevalence of 1·4%, but assessment of age-specific prevalence or IFRs is not feasible given the small sample, low prevalence, and lack of data on case outcomes.
Jersey,<br>United Kingdom[93]	This study collected samples from 629 households comprising 1,062 individuals and estimated seroprevalence at 4·2% (CI 2·9 to 5·5%), indicating that about 3,300 Jersey residents have been infected. Jersey has had 30 COVID-19 fatalities (as of July 15), and hence the overall IFR is about 1% (similar to that of NYC). However, the seroprevalence sample is too small to facilitate accurate assessments of age-specific IFRs; for ages 55+, there were 258 samples and 12 positive cases,

Lisbon, Portugal[94]	This study analyzed specimens from 300 hospital patients and healthcare workers, 2500 university staff, and 187 adult volunteers and found that the ability to detect SARS-CoV-2 antibodies remained robust for five months in a large proportion of previously virus-positive screened subjects.
Louisville, Kentucky, USA[95]	This study analyzed specimens from 2237 individuals, including 509 who responded to mailed invitations and 1728 who volunteered after hearing about the study via news or social media, and found raw seroprevalence of 4·1% (CI: 3·2–5·1%). Age-specific results were not reported as of September 17.
Miami-Dade County, Florida, USA[96]	This study analyzed samples from 2,357 individuals in April and obtained 65 positive IgG results; an additional 275 individuals were tested in June with 4 positive results. Test-adjusted seroprevalence estimates and confidence intervals have not been published as of September 17.
New York City, New York, USA[97]	This study analyzed seroprevalence using specimens from four groups of patients (Cardiology, OB/GYN, Oncology, and Surgery) starting in mid-February. For the final week of the study (April 19), positive results were obtained for 47 of 243 patients; that seroprevalence estimate of 19·3% is well-aligned with the results of the New York Department of Health study. However, the sample size of this cohort is too small for assessing age-specific IFRs.
Neustadt-am-Rennsteig, Germany[98]	This study analyzed seroprevalence of 626 residents (71% of the population of this municipality) and estimated seroprevalence of 8·4% (52 positive cases). However, this sample size is too small for assessing age-specific IFRs.
New Orleans, Louisiana, USA[99]	This study analyzed seroprevalence in a random sample of 2,640 participants and obtained a seroprevalence estimate of 6·9% and an IFR of 1·6% (CI 1·5 to 1·7%). The study did not report on age-specific seroprevalence or IFRs.
New York City, New York, USA[100]	This study estimated age-specific infection fatality rates in New York City during spring 2020 using case, mortality, and mobility data. No seroprevalence data was utilized.
Norbotten, Sweden[101]	This study analyzed a randomly-selected sample of 425 adults and obtained 8 positive results; the test-adjusted seroprevalence was 1·9% (CI: 0·8–3·7%). However, only 2 positive results were for ages 30-64 and 2 positive results for ages 65+, so age-specific prevalence and IFRs cannot be reliably estimated.
Norway[102]	Norwegian Institute of Public Health collected 900 residual serum specimens from nine laboratories from various regions of Norway and obtained test-adjusted seroprevalence of 1·0% (CI: 0.1–2·4%). The study found 4 positive results out of 372 specimens for adults ages 25–59 and 2 positive results out of 206 specimens for adults ages 60+. The authors noted that *"these results should be interpreted with caution"* due to the limited size of the sample.
Occitania, France[103]	This study analyzed samples from 613 individuals *"exposed to the virus to varying extents mimicking the general population in Occitania"* and found seroprevalence of 1·3% (CI: 0·6–2·6%). The study did not report any age-specific data.
Oklahoma, USA[104]	The Oklahoma Department of Health publishes weekly data on raw seroprevalence using samples collected from labs within the state, but its reports do not include test-adjusted estimates, confidence intervals, or age-specific results.
Oslo, Norway[105]	As of August 12, this ongoing study had analyzed specimens from 3250 participants in the Norwegian Mother, Father and Child Survey (MoBa) and found seroprevalence of "less than 2 percent." No confidence intervals or age-specific results were reported.
Pima County, Arizona, USA[106]	This study analyzed specimens from 5882 self-recruited members of the local community and found 60 specimens with neutralizing antibodies (1·0%). Age-specific seroprevalence was not reported.
Rhode Island, USA[107]	This study invited 5000 randomly-selected households, collected samples from "roughly 10 to 15 percent" who agreed to participate, and obtained seroprevalence of 2·2% (CI: 1·1–3·9%). No age-specific results have been reported as of September 17.
Riverside County, California, USA[108]	This study tested a randomized sample of 1,726 residents during July and found raw seroprevalence of 5·9%. The press release (issued on July 27) indicated that the results "are still being analyzed"; no test-adjusted seroprevalence results or age-specific findings have been reported as of September 17.
San Francisco Mission District, California[109]	This study analyzed active infections and seroprevalence of 3,953 residents in a densely population majority Latinx neighborhood in downtown San Francisco. Positive seroprevalence in older adults was very low (22 out of 3,953) and hence too small for assessing age-specific IFRs.
San Miguel County, Colorado, USA[110]	The San Miguel County Health Department assessed seroprevalence in March and April using samples from 5,283 participants (66% of county residents). Raw prevalence was very low (0·53%), with only 3 confirmed positive results for adults ages 60 years and above.

Slovenia[111]	Researchers at the University of Ljubljana assessed seroprevalence using an IgG ELISA test for a random sample of 1,318 participants on April 20 to May 3. Test-adjusted prevalence was 0·9% (CI: 0 to 2·1%), indicating that the sample may have included only 10 infected individuals; no age-specific results were reported.
South-East England[112]	This study collected samples from 481 participants of the TwinsUK cohort and obtained 51 positive results (raw prevalence of 12%). No age-specific results were reported.
Stockholm, Sweden[113]	This study did not directly assess prevalence but produced estimates of IFR for two age groups (ages 0-69 and 70+) using a novel methodology linking live virus tests, reported cases, and mortality outcomes. The estimated IFR was 4·3% for ages 70+.
Stockholm Districts, Sweden[114]	This study analyzed samples from 213 randomly selected individuals in two residential areas of Stockholm on June 17–18 and found markedly different seroprevalence rates of 4·1% and 30%, respectively. No age-specific results were reported.
Stockholm Region, Sweden[115]	Stockholm County began offering antibody testing on a free walk-up basis. As of July 20, 166,431 antibody tests had been performed, of which 17·7% were positive. No demographic data or test-adjusted seroprevalence results had been reported as of September 17.
Miyagi, Osaka, and Tokyo, Japan[116]	This study collected samples from randomly-selected residents of three cities on June 1-7 and used two IgG test kits (Abbott and Roche); results were deemed “positive” only if confirmed by both tests Estimated seroprevalence was 0·1% in Tokyo (2 positive results from 1,971 specimens), 0·17% in Osaka (5 positive results from 2,970 specimens), and 0·03% in Miyagi (1 positive result from 3,009 specimens). No age-specific prevalence estimates were reported.
United States[117]	Seroprevalence estimates are reported in the U.S. CDC’s weekly COVID-19 surveillance summary using data collected by 85 state and local public health laboratories. These reports include age-specific seroprevalence but no details regarding sample selection, test characteristics, or confidence intervals and hence could not be used in our metaregression.
Utsunomiya, Japan[118]	This study tested a random sample of 742 participants and found 3 confirmed positive results among 463 adults ages 18 to 65 years; the test-adjusted prevalence for that age group was 0·65% (CI: 0·13–1·8%). No positive results were obtained for the sample of 181 adults ages 65+ years.
Virginia, USA[119]	Virginia Department of Health collected specimens from a random sample of 3113 participants ages 16+ during early June and estimated prevalence of 2·4%. No confidence intervals or age-specific results had been released as of September 17.
Vo, Italy[120]	Vo’ is a municipality of 3,300 people, nearly all of whom (87%) participated in an infection survey in late February. However, there were only 54 infections among people ages 50+, so assessing age-specific IFRs is not feasible.
Washoe County, Nevada, USA[121]	This study collected samples from 234 individuals on June 9-10 and obtained 5 positive IgG results. No age-specific results were reported.
Winston-Salem, North Carolina, USA[122]	This ongoing study has been collecting specimens from a representative sample of area residents since mid-April, and raw prevalence was characterized as *“about 10%.”* On July 28 the researchers reported that the test was not sufficiently sensitive and that a new test would be deployed henceforth.
Zurich, Switzerland[123]	This study analyzed specimens from 578 individuals, including 90 with prior confirmed COVID-19 infections, 177 with positive patient contacts, and 311 who were randomly selected residents of Zurich. Seroprevalence in the randomly-selected group was estimated at 3·9% using the optimized test method.

H.2: Studies excluded due to accelerating outbreak

Location	Date	Cumulative fatalities in thousands		Change (%)
		Study midpoint	4 weeks later	
Los Angeles, California, USA[124]	April 10-11	0·265	1·468	454
New York City, New York, USA[10]	March 23-April 1	1·066	14·261	1238

H.3 Studies excluded due to non-representative samples

H.3.1 Active recruitment of participants

Location	Description
Luxembourg[125]	Of the 35 participants who tested positive, 19 had previously interacted with a person who was known to be infected or had a prior test for SARS-CoV-2.
Boise, Idaho[126]	This study was promoted during a "Crush the Curve" publicity campaign and required participants to sign up for a test.
Santa Clara, California, USA[127]	Participants were recruited via social media and needed to drive to the testing site. Stanford Medicine subsequently released a statement indicating that the study was under review due to concerns about potential biases.[128]
Frankfurt, Germany[129]	This study was conducted at an industrial worksite. Among the 5 seropositive participants, 3 had prior positive tests or direct contact with a known positive case.

H.3.2 Studies of hospitals, urgent care clinics, and dialysis centers

Location	Description
Brooklyn, New York, USA[130]	This study used samples from an outpatient clinic and yielded a much higher infection rate than other seroprevalence studies of the New York metropolitan area.
Kobe, Japan[131]	This study tested for IgG antibodies in 1,000 specimens from an outpatient clinic and found 33 positive cases. However, the study did not screen out samples from patients who were seeking treatment for COVID-related symptoms. Moreover, the study reported raw prevalence and confidence interval but did not report statistics adjusted for test characteristics. The manufacturer (ADS Biotec / Kurabo Japan) has indicated that this test has specificity of 100%, based on a sample of 14 pre-COVID specimens, but that specificity has not been evaluated by any independent study. The authors concluded by noting the selection bias and recommended that *"further serological studies targeting randomly selected people in Kobe City could clarify this potential limitation."*
Tokyo, Japan[132, 133]	The authors of this study specifically cautioned against interpreting their results as representative of the general population. In particular, the sample of 1,071 participants included 175 healthcare workers, 332 individuals who had experienced a fever in the past four months, 45 individuals who had previously taken a PCR test, and 9 people living with a COVID-positive cohabitant. The study obtained a raw infection rate of 3·8%, but the rate is only 0·8% if those subgroups are excluded.
United States[134]	This study analyzed residual plasma of 28503 randomly selected adult patients receiving dialysis in July 2020. Seroprevalence for the tested sample was 8·0% (CI: 7·7–8·4%), However, prevalence of dialysis patients can diverge markedly from that of the general population; for example, this sudy finds a seropositive rate of 34% for patients residing in New York state, nearly three times higher than the 14% seroprevalence rate obtained using a random sample of that state.[11]
Zurich, Switzerland[135]	This study analyzed two distinct set of samples: (i) blood donors and (ii) hospital patients. Nearly all blood donors were ages 20 to 55, so that sample is not useful for assessing age-specific IFRs for older adults. The sample of hospital patients was not screened to eliminate cases directly related to COVID-19, so that sample may not be representative of the broader population, e.g., inhabitants of the city of Zurich constituted a relatively large fraction of seropositive results compared to residents from the rest of the canton of Zurich. The study found an overall IFR of 0·5% similar to that of Geneva.

H.3.3 Studies of blood donors

Location	Description
Apulia, Italy[136]	This study assessed specimens from a sample of 904 healthy blood donors at a transfusion center in southeastern Italy and obtained 9 positive results (0·99%).
Canada[137]	Canadian Blood Services analyzed 37737 specimens from blood donors collected between 9 May and 18 June 2020 and found 275 positive results. Test-adjusted seroprevalence was 0·7% (CI:0·60–0·79%).
Denmark[138]	This study assessed specimens from a sample of 20640 Danish blood donors and calculated a test-adjusted prevalence of 1·9% (CI:0·8–2·3). Unfortunately, the antibody test used in this study was subsequently identified as unreliable, and the Danish government returned all remaining test kits to the manufacturer.[139]
England[140]	Public Health England has conducted ongoing surveillance of seroprevalence using specimens from healthy adult blood donors. For example, in 7694 samples tested during May (weeks 18-21), the test-adjusted prevalence was 8·5% (CI: 6·9–10%).
Germany[141]	This study assessed residue sera from 3186 regular blood donors collected during March 9–June 3 and obtained 29 positive results (raw prevalence 0·9%). The authors stated: *"It should be emphasized that the preselection of blood donors as a study cohort is accompanied by limitations regarding representation of population."*
Lombardy, Italy[142]	This study assessed specimens from 390 blood donors residing in the Lodi red zone collected on April 6 and found a raw seroprevalence rate of 23%.
Milan, Italy[143]	This study assessed specimens from a random sample of 789 blood donors over the period from February 24 (at the start of the outbreak) to April 8.
Netherlands[144]	This study assessed specimens from 7361 adult blood donors collected on April 1-15 and found seroprevalence of 2·7%.
Rhode Island, USA[145]	This study assessed specimens from 2008 blood donors collected during April 27–May 11 and found seroprevalence of 0·6%.
Scotland[146]	This study assessed specimens from 3500 blood donors collected between March 17 and May 19. The authors noted that the resulting estimates of seroprevalence *"are complicated by non-uniform sampling...based on the locations where weekly donations took place...[and] further confounded by the absence of samples from individuals below age 18 and individuals over age 75."*
San Francisco, California, USA[147]	This study assessed specimens from 1000 blood donors that were collected during March and found one positive result (raw prevalence 0·1%).
United States[148]	This study analyzed residual sera from 252882 U.S. blood donors obtained between June 1 and July 31 and found an overall seroprevalence of 1·83%.
United States[149]	This study analyzed residual sera from 953926 U.S. blood donors obtained between June 15 and August 23 and found seropositivity of 1·82% (CI: 1·79–1·84%).

H.3.4 Studies of elementary schools

Location	Description
Oisie, France[150]	This sample of 1,340 participants included elementary school teachers, pupils, and their families. Only two individuals in the sample were ages 65 years and above.
Saxony, Germany[151]	This study analyzed specimen samples from students and teachers at thirteen secondary schools in eastern Saxony and found very low seroprevalence (0·6%).
Southwest Germany[76]	This study analyzed specimens from 2482 children (ages 1–10 years) and one of their parents collected between 22 April and 15 May 2020. Seroprevalence for children was 0·6% (CI: 0·3–1·0%), while the prevalence among their parents was 1·8% (CI: 1·2–2·4%).
Zurich, Switzerland[152]	This study collected samples from 2585 school children ages 6–16 years and found seroprevalence of 2·8% (CI: 1·6–4·1%).

H.3.5 Life insurance applicants

Location	Description
United States[153]	This study analyzed specimens for 50130 consecutive life insurance applicants whose blood samples were collected for insurance underwriting purposes between 12 May and 25 June 2020. The study found 1520 positive results, that is, raw seroprevalence of 3·0%. The study did not find significant differences in prevalence across three age groups (18–40, 41–60, and 61–85 years).

H.3.5 Close contacts of positive cases

Location	Description
Lombardy, Italy[154]	This study used a database of 62881 contacts of COVID-19 cases and conducted RT-PCR tests and antibody screening on 5484 individuals. The study reported that 2824 individuals had positive tests (51·5% of the sample), of which 62 individuals subsequently died with a COVID-19 diagnosis.

H.4 Exclusion of observations with seroprevalence indistinguishable from zero

Note: The metaregression analysis excludes observations for which either (a) the lower bound of the 95% confidence interval equals zero, and hence the upper bound of the IFR is not well defined; or (b) the lower bound of the 95% confidence interval is less than the observed COVID-19 mortality rate for that age group, implying an upper bound for the IFR that exceeds 100%.

H.4.1 Exclusion of observations from European seroprevalence studies

			95% Confidence Interval (%)	
**Location**	**Age Group**	**Prevalence (%)**	**Lower Bound**	**Upper Bound**
Hungary[17]	14–39	0·1	0·0	0·5
	40–64	0·3	0·0	0·6
	65+	0·4	0·0	0·9
Portugal[18]	20–39	0·9	0·1	5·3

H.4.2 Exclusion of observations from U.S. seroprevalence studies

			95% Confidence Interval (%)	
**Location**	**Age Group**	**Prevalence (%)**	**Lower Bound**	**Upper Bound**
Atlanta, Georgia, USA[155]	0–17	0	0	1·0
	65+	0·7	0·1	4·5
Connecticut, USA[10]	0–18	0·8	0	2·9
Louisiana, USA[10]	0–18	2·8	0	11·5
Miami, Florida, USA[10]	0–18	2·4	0	7·8
Minneapolis, Minnesota, USA[10]	0–18	5·8	0	14·3
	50–59	0·7	0	2·8
	60+	1·0	0	3·2
Missouri, USA[10]	0–18	1·4	0	4·1
Oregon, USA[156]	0–17	0	0	10·4
	18–49	0	0	0·7
	50–64	0·1	0	1·0
	65+	1·4	0·1	2·8
Philadelphia, Pennsylvania, USA[10]	0–18	2·2	0	6·9
	50–64	0·8	0	2·8
	65+	1·6	0·3	3·5
Salt Lake City, Utah, USA[14]	65+	0·6	0	1·4
San Francisco, California, USA[10]	0–18	1·7	0	7·7
	19–49	1·1	0	2·6
	50–64	0·7	0	2·4
Seattle, Washington, USA[10]	0–18	0·7	0	2·5

H.5 Exclusion of observations with no observed fatalities

Location	Age Group	Population *(millions)*	**Infections** *(thousands)* Estimate	95% confidence interval
Geneva	5–19	0·796	7·300	4·300–11·200
Hungary	0–14	1·391	7·795	3·758–11·971
Portugal	0–9	0·841	17·663	6·729–45·418
	10–19	1·015	21·318	8·121–55·834
Australia	0–39	13·533	2·800	4·200–12·600
Iceland	0–29	0·136	0·554	0·407–0·608
Korea	0–29	15·623	15·180	6·939–21·685
Lithuania	0–39	1·198	1·845	0·843–2·635
New Zealand	0–59	3·751	3·726	1·876–5·627

Note: This table shows observations for relatively young age groups in locations where no COVID-19 fatalities were recorded for that age group and hence the implied value of the infection fatality rate is at its lower bound of zero and its confidence interval cannot be precisely determined.

Appendix I: Seroprevalence Rates for Studies Included in Meta-Analysis

I.1 European seroprevalence studies with representative samples

Location	Dates in 2020	Age Group, years	Population, millions	Prevalence (%)	95% Confidence Interval (%)
England[157]	April 16–July 3	0–17	12·023	9·2	6·2–12·2
England[15]	June 20–July 13	18–24	4·747	7·9	7·3–8·5
		25–34	7·609	7·8	7·4–8·3
		35–44	7·147	6·1	5·7–6·6
		45–54	7·623	6·4	6·0–6·9
		55–64	6·782	5·9	5·5–6·4
		65–74	5·576	3·2	2·8–3·6
		75+	4·778	3·3	2·9–3·8
France[25]	May 4–24	0–39	11·669	12·0	10·6–13·5
		40–49	3·124	12·7	11·4–14·0
		50–59	3·097	6·5	5·5–7·4
		60–69	2·691	4·5	3·8–5·3
		70+	3·209	3·4	2·7–4·1
Ireland[26]	June 22–July 16	15–44	1·971	1·5	0·3–2·7
		45–64	1·218	1·2	0·3–2·1
Italy[16]	July 6–27	0–19	10·859	1·8	1·3–2·4
		20–29	6·201	1·7	1·3–2·0
		30–49	16·317	2·0	1·7–2·4
		50–59	9·352	2·7	2·3–3·1
		60–69	7·337	2·2	1·7–2·5
		70+	10·278	2·1	1·7–2·5
Netherlands[158]	April 1–17	0–49	10·053	3·5	2·5–5·2
		50–59	2·524	4·3	3·2–5·8
		60–69	2·130	3·5	2·5–5·0
		70–79	1·592	3·0	1·7–5·3
		80+	0·837	2·8	0·9–7·3
Portugal[18]	May 21–July 8	0–9	0·841	2·2	0·9–6·0
		10–19	1·015	2·4	0·9–6·1
		20–39	2·289	0·9	0·1–5·3
		40–59	3·057	2·6	1·0–6·6
		60+	2·995	2·7	1·2–5·4
Spain[41]	May 18–June 1	0–9	4·284	2·4	1·1–4·8
		10–19	4·955	2·9	2·0–4·1
		20–29	4·883	3·4	2·4–4·8
		30–39	5·902	3·1	2·2–4·1
		40–49	7·938	3·6	2·8–4·5
		50–59	7·046	3·7	2·8–4·7
		60–69	5·340	3·4	2·5–4·5
		70+	6·939	3·1	1·9–5·0
Geneva, Switzerland[39]	April 13–May 8	5–19	0·080	9·2	5·4–14·1
		20–49	0·219	13·1	9·8–17·0
		50–64	0·099	10·5	7·3–14·1
		65+	0·084	6·8	3·8–10·5

I.2 European and Canadian seroprevalence studies with convenience samples

Location	Dates in 2020	Age Group *(years)*	Population *(millions)*	Prevalence (%)	95% Confidence Interval (%)
Belgium[33]	April 20–26	0–24	3·229	6·7	4·7–9·6
		25–44	2·957	6·6	4·7–9·2
		45–64	3·081	6·9	5·2–9·2
		65–74	1·147	4·6	2·6–8·0
		75–84	0·691	7·8	4·7–13·0
		85+	0·327	14·7	9·9–21·8
Ontario[159]	June 5–30	0–19	3·142	0·8	0·3–1·4
		20–59	7·977	1·0	0·7–1·3
		60+	3·448	1·6	1·1–2·1
Sweden[46]	April 27–May 24	0–19	2·321	5·7	4·5–7·0
		20–49	3·861	6·5	5·2–7·8
		50–69	2·390	4·8	3·6–6·0
		70+	1·526	3·1	2·1–4·1

I.3 U.S. seroprevalence studies with representative samples

Location	Dates in 2020	Age Group *(years)*	Population *(millions)*	Prevalence (%)	95% Confidence Interval (%)
Atlanta, USA[155]	April 28–May 3	0–17	0·402	0·0	0·0–1·0
		18–49	0·867	3·3	1·6–6·4
		50–64	0·328	4·9	1·8–12·9
		65+	0·226	0·7	0·1–4·5
Indiana, USA[19]	April 25–29	0–39	3·546	2·7	1·5–3·9
		40–59	1·674	2·8	1·4–4·2
		60+	1·512	1·3	0·6–2·0
New York, USA[11]	April 23	0–19	4·898	14·6	13·1–16·1
		20–39	5·409	14·6	13·1–16·1
		40–49	2·356	15·3	13·7–17·0
		50–59	2·623	16·0	14·6–17·5
		60+	4·544	12·1	11·2–13·1
Salt Lake City, USA[160, 161]	May 4–June 10	0–44	1·544	1·2	0·4–2·5
		45–64	0·427	0·9	0·2–2·1
		65+	0·223	0·6	0·0–1·4

I.4 U.S. Seroprevalence Studies with Convenience Samples

Location	Dates in 2020	Age Group *(years)*	Population *(millions)*	Prevalence (%)	95% Confidence Interval (%)
Connecticut, USA[10]	April 26–May3	0·830	0–19	0·8	0·0–2·9
		1·341	20–49	6·1	3·1–9·3
		0·519	50–59	8·1	4·8–
		0·876	60+	4·2	2·3–6·0
Louisiana, USA[10]	April 1–8	1·146	0–18	2·8	0·0–11·5
		1·879	19–49	7·4	4·7–10·0
		0·587	50–59	8·3	4·5–11·9
		1·037	60+	4·4	1·5–8·0
Miami, USA[10]	April 6–10	1·341	0–19	2·4	0·0–7·8
		2·513	20–49	0·9	0·2–2·2
		1·272	50–59	2·0	0·3–4·0
		1·203	60+	3·0	1·7–4·5
Minneapolis, USA[10]	April 30–May 12	0·966	0–18	5·8	0·0–14·3
		1·610	19–49	2·3	0·8–4·2
		0·513	50–59	0·7	0·0–2·8
		0·809	60+	1·0	0·0–3·2
Missouri, USA[10]	April 20–26	1·527	0–19	1·4	0·0–4·1
		2·348	20–49	3·4	1·4–5·5
		0·796	50–59	2·0	0·5–3·8
		1·466	60+	3·2	1·9–4·6
Philadelphia, USA[10]	April 13–25	0·944	0–18	2·2	0·0–6·9
		1·707	19–49	5·9	2·4–9·8
		1·268	50–59	0·8	0·0–2·8
		0·825	60+	1·6	0·3–3·5
San Francisco, USA[10]	April 23–27	1·649	0–18	1·7	0·0–7·7
		2·960	19–49	1·1	0·0–2·6
		1·262	50–59	0·7	0·0–2·4
		1·023	60+	0·9	0·2–2·5
Seattle, USA[10]	March 23–April 1	1·009	0–19	0·7	0·0–2·5
		1·332	20–39	1·3	0·7–2·3
		1·115	40–59	0·9	0·3–1·9
		0·870	60+	1·7	0·9–2·7

I.5 Prevalence in countries with comprehensive tracing programs

Location	Dates in 2020	Population, millions	Age Group, years	Prevalence (%)	95% Confidence Interval (%)
Australia[60]	February 1–June 12	13·533	0–39	0·06	0·03–0·09
		6·414	40–59	0·06	0·04–0·10
		2·651	60–69	0·09	0·05–0·13
		1·846	70–79	0·08	0·04–0·12
		1·055	80+	0·05	0·03–0·07
Iceland[31]	February 1–June 15	0·136	0–29	0·4	0·3–0·5
		0·132	30–59	1·1	0·8–1·6
		0·038	60–69	0·5	0·3–1·0
		0·023	70–79	0·3	0·27–1·3
		0·013	80+	0·2	0·1–2·5
Korea[61]	February 1–May 17	15·623	0–29	0·11	0·05–0·17
		7·080	30–39	0·08	0·04–0·12
		8·219	40–49	0·06	0·03–0·09
		8·477	50–59	0·07	0·03–0·10
		6·454	60–69	0·05	0·03–0·08
		3·560	70–79	0·05	0·03–0·07
		1·856	80+	0·06	0·03–0·09
Lithuania[62]	February 1–June 18	1·198	0–39	0·15	0·07–0·22
		0·356	40–49	0·18	0·10–0·29
		0·421	50–59	0·17	0·10–0·33
		0·353	60–69	0·13	0·08–0·20
		0·223	70–79	0·09	0·05–0·14
		0·172	80+	0·13	0·07–0·19
New Zealand[63]	February 1–July 9	3·751	0–59	0·10	0·05–0·15
		0·522	60–69	0·07	0·04–0·10
		0·362	70–79	0·04	0·02–0·07
		0·187	80+	0·04	0·02–0·06

I.6 Prevalence estimates of large-scale studies used in out-of-sample analysis

Location	Dates in 2020	Population, millions	Age Group, years	Prevalence (%)	95% Confidence Interval (%)
England (ONS)	April 26–July 26	25·051	15–49	6·1	4·9–7·5
		15·738	50–69	4·8	4.0–5·8
		8·782	70+	3·9	3·0–5·2
France[162]	April 6–12	0-9	7·527	5·9	1·6–10·2
		10-19	7·883	3·5	0·7–6·4
		20-29	7·371	7·0	3·8–10·2
		30-39	8·011	3·4	1·0–5·8
		40-49	8·326	7·7	4·6–10·9
		50-59	8·635	9·7	6·4–13·1
		60-69	7·765	10·0	6·5–13·5
		70–79	5·728	5·9	3·1–8·7
		80+	4·027	7·3	4·2–10·3
Great Britain (U.K. Biobank)	May 27–July 6	23·562	0–29	10·8	9·4–12·3
		8·641	30–39	8·2	7·2–9·3
		8·180	40–49	7·2	6·2–8·4
		8·810	50–59	7·1	6·2–8·0
		6·928	60–69	6·4	5·6–7·2
		8·782	70+	5·4	4·7–6·1
Utah, USA (CDC)	April 20–May 3	1·228	19–44	1·8	0·6–3·5
		0·632	45–64	2·9	0·9–5·2
		0·366	65+	2·7	0·9–5·0

I.7 Prevalence estimates of small-scale studies used in out-of-sample analysis

Location	Dates in 2020	Population,	Age Group, years	Prevalence (%)	95% Confidence Interval (%)
Diamond Princess cruise ship	Feb 1–March 7	**1150**	0–49	8·3	NA
		**398**	50–59	14·8	NA
		**923**	60–69	19·2	NA
		**1015**	70–79	23·1	NA
		**216**	80+	25·0	NA
Castiglione d'Adda, Italy	May 18–25	**3052**	15–64	19·1	14·9–23·2
		**538**	65–74	31·3	25·4–37·3
		**401**	75–84	36·6	28·3–44·9
		**149**	85+	42·1	31·1–53·1

Supplementary Appendix J: Assessment of risk of bias for included studies

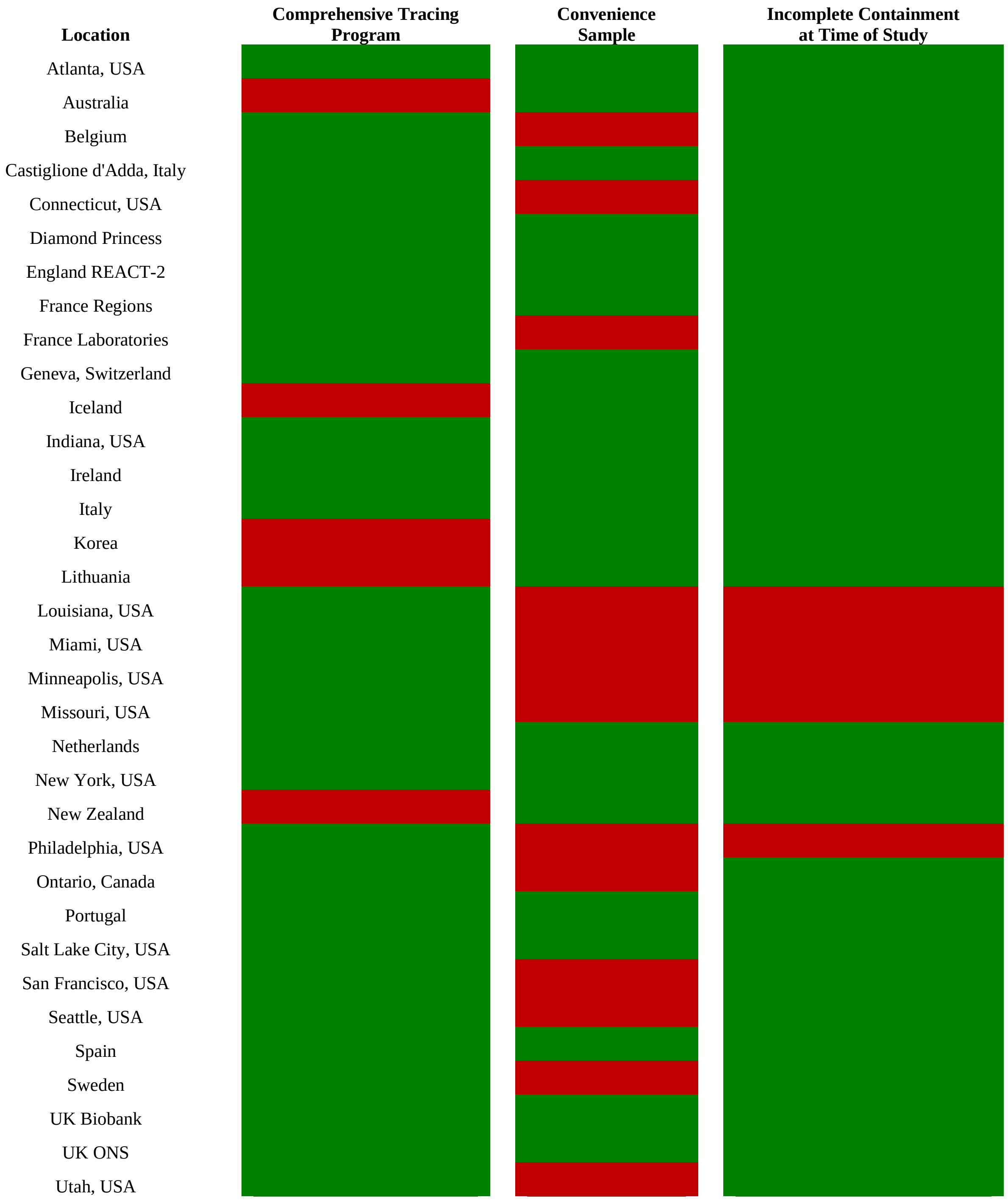

Supplementary Appendix K: Assessment of Publication Bias

(1) Regression-based Egger test for small-study effects
Random-effects model estimated using REML
H0: β = 0; no small-study effects
β = 0.04, SE(β) = 0.136,
z = 0.27, Prob > |z| = 0.7882

(2) Nonparametric trim-and-fill analysis of publication bias
Linear estimator, imputing on the right
Number of studies = 105
Model: Random-effects
Method: REML
Observed = 104, Imputed = 1
Effect Size and 95% Confidence Interval
Observed: 0.000 (-0.009, 0.009)
Observed + Imputed: 0.000 (-0.009, 0.009)

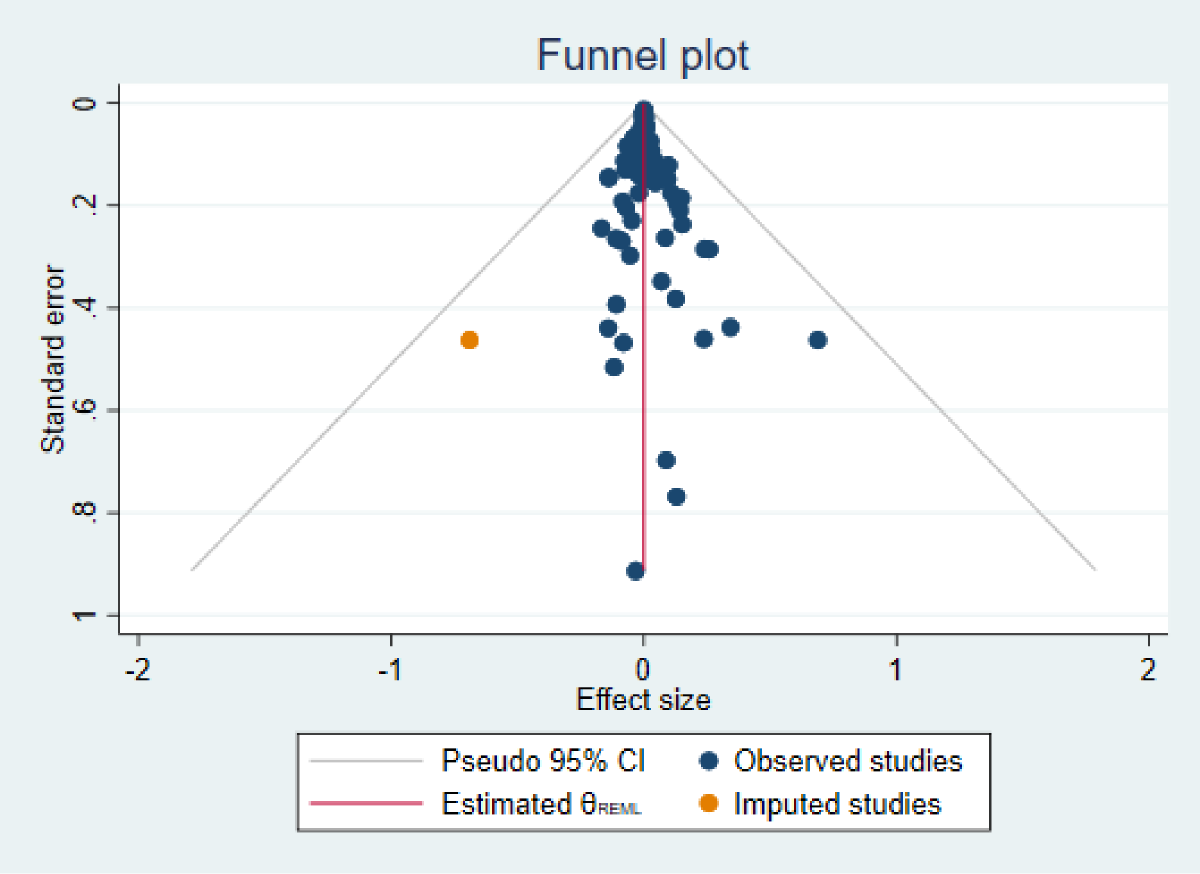

Supplementary Appendix L: Sensitivity analysis of metaregression results

L.1: Stability of coefficients across age groups

Age Category	# Metaregression Observations	Intercept (95% CI)	Slope Coefficient (95% CI)
Age < 35 Years	24	-3·46 (-3·81, -3·11)	0·065 (0·050, 0·080)
35 ≤ Age ≤ 60 Years	38	-3·13 (-3·69, -2·57)	0·048 (0·037, 0·060)
Age > 60 Years	41	-3·27 (-3·61, -2·93)	0·053 (0·038, 0·067)
All Ages	104	-3·27 (-3·61, -2·93)	0·052 (0·038, 0·067)

Parameter Stability Test: H_0 = stability of intercept and slope coefficient across age categories
F Statistic (4,102) = 2·13, P-value = 0·083 ***(i.e., H_0 is not rejected at 95% confidence level)***

L.2: Exclusion of open-ended top age groups

Age Category	# Metaregression Observations	Intercept (95% CI)	Slope Coefficient (95% CI)
Benchmark Regression	104	-3·27 (-3·61, -2·93)	0·053 (0·038, 0·067)
Exclude 60+ and 65+	93	-3·26 (-3·41, -3·11)	0·052 (0·049, 0·055)
Exclude 60+, 65+, 70+, and 75+	88	-3·22 (-3·37, -3·07)	0·051 (0·048, 0·054)
Exclude All Top Age Groups	81	-3·22 (-3·39, -3·06)	0·051 (0·048, 0·054)

Supplementary Appendix M: Out-of-sample analysis of metaregression results

Study	Description
Castiglione d'Adda, Italy[163]	This study assessed seroprevalence in a random sample of 509 residents of the municipality of Castiglione d'Adda, the location of the first COVID-related fatality in Italy. Specimens were collected on May 18–25. This study is included in our meta-analysis but not in our metaregression because this municipality is covered by a nationwide seroprevalence study of Italy.[16]
Diamond Princess Cruise Ship[164]	This ship was carrying 3,711 passengers and crew. RT-PCR tests indicated that 619 individuals had been infected prior to the ship's debarkation on March 7, and 14 individuals subsequently died due to COVID-related causes.
France Laboratories[162]	Santé Publique France analyzed 3084 specimens of residual sera from two French laboratories (Cerba and Eurofins Bimnis) obtained during the week of 6–12 April, using the LuLISA-N test developed at the Institut Pasteur. Our metaregression includes a larger study of a representative sample of the French population in three regions (Grand Est, Ile de France, and Nouvelle Aquitaine) that accounted for about two-thirds of COVID-19 cases and fatalities during spring 2020. Consequently, this study is included in our meta-analysis but not in our metaregression to avoid pitfalls of nested or overlapping samples.
U.K. Biobank[165]	This study of Great Britain assessed seroprevalence using specimens collected from a demographically balanced panel of 17,776 participants on May 27 to July 6. Our metaregression includes a much larger seroprevalence study of the English population.[15] Consequently, this study is included in our meta-analysis but not in our metaregression to avoid pitfalls of nested or overlapping samples.
U.K. Office of National Statistics[13]	The U.K. Office for National Statistics (ONS) regularly reports estimates of seroprevalence from specimens provided for routine testing using an IgG ELISA test conducted by research staff at the University of Oxford. On August 18 the ONS reported age-specific results for the cumulative sample of 4840 specimens received from 26 April to 26 July and indicated that these results were broadly consistent with the findings of the UK REACT-2 study (which utilized a much larger sample).
Utah, USA[10]	This study analyzed commercial lab specimens from 1132 individuals collected during April 20–May 3. This study is not included in our meta-analysis because a subsequent study analyzed a much larger randomized sample of 6527 residents of the Salt Lake City metropolitan area during May 4–June 10.[160] As of May, that metro area accounted for nearly 90% of COVID-related fatalities in Utah.

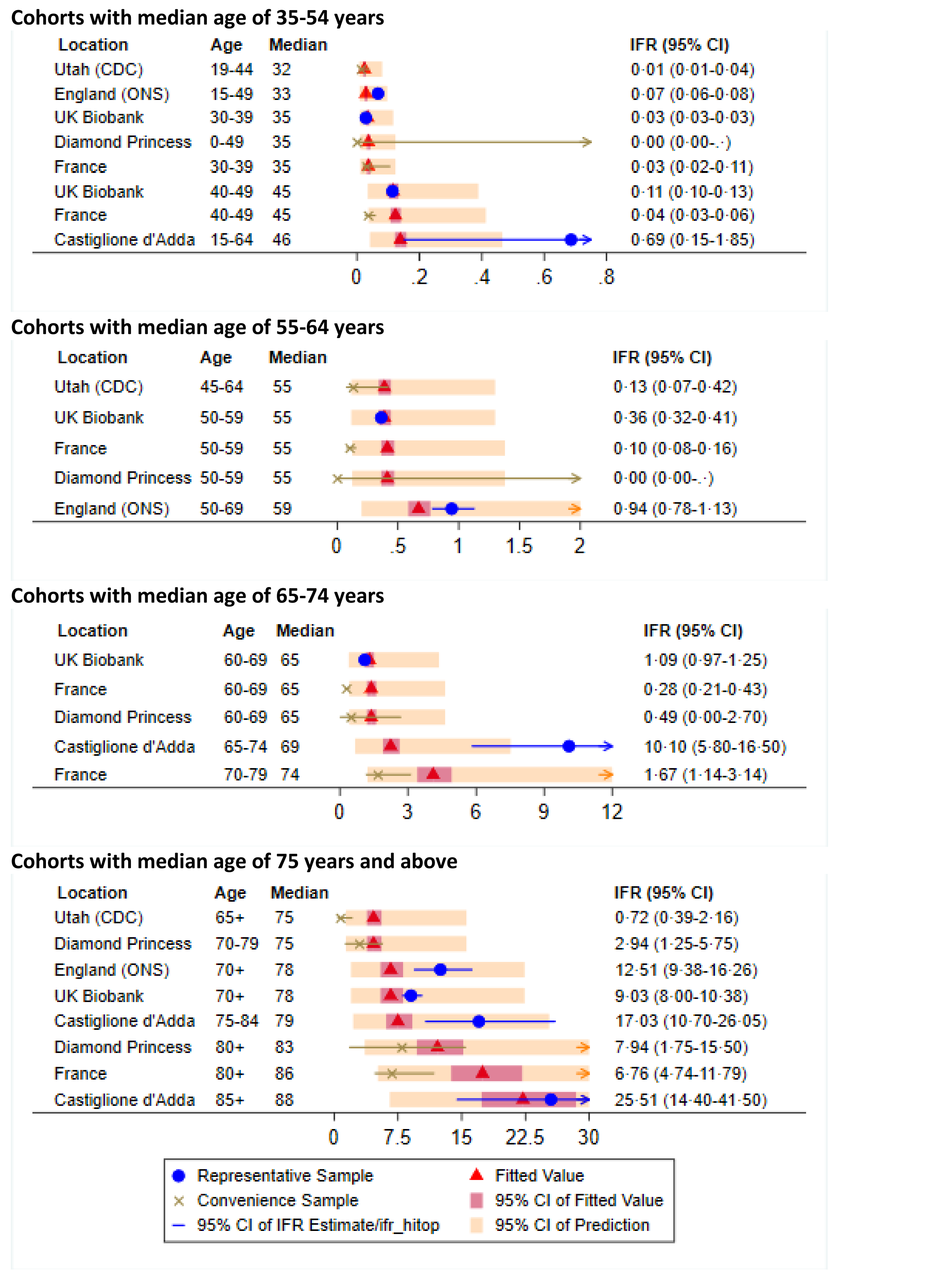
Cohorts with median age of 35-54 years
Location Age Median IFR (95% CI)
Utah (CDC) 19-44 32 0·01 (0·01-0·04)
England (ONS) 15-49 33 0·07 (0·06-0·08)
UK Biobank 30-39 35 0·03 (0·03-0·03)
Diamond Princess 0-49 35 0·00 (0·00-.·)
France 30-39 35 0·03 (0·02-0·11)
UK Biobank 40-49 45 0·11 (0·10-0·13)
France 40-49 45 0·04 (0·03-0·06)
Castiglione d'Adda 15-64 46 0·69 (0·15-1·85)
0 .2 .4 .6 .8
Cohorts with median age of 55-64 years
Location Age Median IFR (95% CI)
Utah (CDC) 45-64 55 0·13 (0·07-0·42)
UK Biobank 50-59 55 0·36 (0·32-0·41)
France 50-59 55 0·10 (0·08-0·16)
Diamond Princess 50-59 55 0·00 (0·00-.·)
England (ONS) 50-69 59 0·94 (0·78-1·13)
0 .5 1 1.5 2
Cohorts with median age of 65-74 years
Location Age Median IFR (95% CI)
UK Biobank 60-69 65 1·09 (0·97-1·25)
France 60-69 65 0·28 (0·21-0·43)
Diamond Princess 60-69 65 0·49 (0·00-2·70)
Castiglione d'Adda 65-74 69 10·10 (5·80-16·50)
France 70-79 74 1·67 (1·14-3·14)
0 3 6 9 12
Cohorts with median age of 75 years and above
Location Age Median IFR (95% CI)
Utah (CDC) 65+ 75 0·72 (0·39-2·16)
Diamond Princess 70-79 75 2·94 (1·25-5·75)
England (ONS) 70+ 78 12·51 (9·38-16·26)
UK Biobank 70+ 78 9·03 (8·00-10·38)
Castiglione d'Adda 75-84 79 17·03 (10·70-26·05)
Diamond Princess 80+ 83 7·94 (1·75-15·50)
France 80+ 86 6·76 (4·74-11·79)
Castiglione d'Adda 85+ 88 25·51 (14·40-41·50)
0 7.5 15 22.5 30
Representative Sample
Convenience Sample
95% CI of IFR Estimate/ifr_hitop
Fitted Value
95% CI of Fitted Value
95% CI of Prediction

Supplementary Appendix N: U.S. scenario analysis

Scenario	Infection Rate by Age (*percent*) All	0-49	50-64	65+	Deaths (*thousands*)	IFR (*percent*)
**Scenario #1:** *uniform prevalence*	20	20	20	20	867	1·3
**Scenario #2:** *protection of vulnerable age groups*	20	26	10	6	348	0·5

Note: These scenarios have the same average infection rate of 20% but with distinct patterns of age-specific prevalence. The metaregression estimates are used to compute total fatalities for each scenario using U.S. population data and predicted age-specific IFRs by single year of age.

Supplementary Appendix O: Infection fatality rate for seasonal influenza

According to the World Health Organization, seasonal influenza mortality is usually well below 0·1% while also noting that "mortality is to a large extent determined by access to and quality of health care."[166] The U.S. Center for Disease Control and Prevention provides annual estimates of the U.S. impact of seasonal influenza based on reporting of symptomatic cases from state and local public health laboratories and other sources. For the winter season of 2018-2019, its preliminary estimate is 35·5 million symptomatic cases and 34 thousand fatalities.[167] A systematic review and meta-analysis of 55 studies found that 25·4% to 61·8% of influenza infections were subclinical, i.e., did not meet the criteria for acute respiratory illness.[168] Using the midpoint of that interval, we estimate that the total U.S. incidence of seasonal influenza during winter 2018-19 was in the range of 45 million to 93 million infections and hence that the population IFR for seasonal influenza was in the range of 0.04% to 0.08%--an order of magnitude smaller than the population IFR for COVID-19.

Supplementary Appendix P: Excess mortality

In some locations, reported deaths may not fully capture all fatalities resulting from COVID-19 infections, especially when a large fraction of such deaths occurs outside of medical institutions. In the absence of accurate COVID-19 death counts, *excess mortality* can be computed by comparing the number of deaths for a given time period in 2020 to the average number of deaths over the comparable time period in prior calendar years, e.g., 2015 to 2019. This approach has been used to conduct systematic analysis of excess mortality in European countries.[169] For example, the Belgian study used in our metaregression computed age-specific IFRs using seroprevalence findings in conjunction with data on excess mortality in Belgium; the authors noted that Belgian excess mortality over the period from March to May coincided almost exactly with Belgium's tally of reported COVID-19 cases.[33]

Supplementary Appendix Q: Comparison of age-specific IFRs

Age	Verity et al. (2020) IFR(%)	Verity et al. (2020) 95% CI	Ferguson et al. (2020) IFR (%)	Metaregression Results IFR	Metaregression Results 95% CI
0-9	0·00161	(0·0002–0·02)	0·002	0·001	(0·0007–0·0013)
10-19	0·00695	(0·001–0·05)	0·006	0·003	(0·002–0·004)
20-29	0·0309	(0·014–0·092)	0·03	0·011	(0·009–0·013)
30-39	0·084	(0·04–0·19)	0·08	0·037	(0·031–0·043)
40-49	0·161	(0·08–0·32)	0·15	0·123	(0·108–0·141)
50-59	0·595	(0·34–1·28)	0·60	0·413	(0·362–0·471)
60-69	1·93	(1·11–3·89)	2·2	1·38	(1·19–1·61)
70-79	4·28	(2·45–8·44)	5·1	4·62	(3·83–5·57)
80+	7·8	(3·80–13·3)	9·3	15·46	(12·2–19·5)

Note: This table compares the metaregression results of this paper with the estimated age-specific IFRs obtained by Verity et al. (2020).[170] Those IFR estimates were subsequently adjusted by Ferguson et al. (2020) to account for non-uniform prevalence, but age-specific confidences were not reported by that study.[171]

Supplementary Appendix R: Comparison of age-specific IFRs and CFRs

Age (years)	IFR (%)	CFR (%)	Ratio
0-29	0·003	0·3	100
30-39	0·037	0·5	14·7
40-49	0·12	1·1	9·2
50-59	0·41	3·0	7·5
60-69	1·4	9·5	7·1
70-79	4·6	22·8	5·1
80+	15·5	29·6	2·0

Note: This table compares the estimated age-specific IFRs from our metaregression with the age-specific case fatality rates (CFRs) of Bonanad et al. (2020).[66]

Supplementary Appendix S: Comorbidities and Demographic Factors

While age and fatality risk are closely related, differences in the age structure of the population and age-specific infection rates surely cannot explain all deviations in IFR across regions and populations. Consequently, the role of co-morbidities and other demographic and socioeconomic factors merits further research that carefully distinguishes between infection risk and IFR.

A recent U.K. study has shown that COVID-19 mortality outcomes are strongly linked to comorbidities such as chronic pulmonary disease, diabetes, and obesity.[172] However, that study specifically warns against drawing causal conclusions from those findings, which may reflect a higher incidence of COVID-19 rather than a higher IFR for individuals with those comorbidities. Indeed, as shown in Table S1, a study of hospitalized U.K. COVID-19 patients found that patient age was far more important than any specific comorbidity in determining mortality risk.[67] For example, the COVID-19 fatality risk for an obese 40-year-old hospital patient was found to be moderately higher than for a non-obese individual of the same cohort but only one-tenth the fatality risk for a non-obese 75-year-old hospital patient.

The high prevalence of comorbidities among COVID-19 patients has been well documented but not compared systematically to the prevalence of such comorbidities in the general population. For example, one recent study of hospitalized COVID-19 patients in New York City (NYC) reported that 94% of those patients had at least one chronic health condition.[173] However, as shown in Table S2, that finding is not particularly surprising given the prevalence of comorbidities among middle-aged and elderly NYC residents. For example, nearly 30% of older NYC adults (ages 60+) are diabetic, while 23% have cardiovascular disease (including hypertension), and 8% have chronic pulmonary diseases—practically identical to the incidence of those comorbidities in the sample of hospitalized COVID-19 patients. Indeed, obesity was the *only* comorbidity that was much more prevalent among hospitalized COVID-19 patients than in the general population of older NYC adults. Nonetheless, obesity is also much more prevalent among lower-income groups who are more likely to live in high-density neighborhoods and work in high-exposure jobs, and hence such data clearly cannot be used to distinguish prevalence vs. severity of COVID-19.

Our meta-analysis has not directly considered the extent to which IFRs may vary with other demographic factors, including race and ethnicity. Fortunately, valuable insights can be garnered from other recent studies. In particular, one recent seroprevalence study of residents of two urban locations in Louisiana found no significant difference in IFRs between whites and Blacks.[99]

Nonetheless, the incidence of COVID-19 mortality among people of color is extraordinarily high due to markedly different infection rates that reflect systematic racial and ethnic disparities in housing and employment. For example, a recent infection study of a San Francisco neighborhood found that 80% of positive cases were Latinx – far higher than the proportion of Latinx residents in that neighborhood.[109] That study concluded as follows: *"Risk factors for recent infection were Latinx ethnicity, inability to shelter-in-place and maintain income, frontline service work, unemployment, and household income less than $50,000 per year."*

Other researchers have reached similar conclusions, attributing elevated infection rates among Blacks and Hispanics to dense housing of multi-generational families, increased employment in high-contact service jobs, high incidence of chronic health conditions, and lower quality of health care.[174]

In summary, while our meta-analysis has investigated the effects of age on IFR for COVID-19, further research needs to be done on how infection and fatality rates for this disease are affected by comorbidities as well as demographic and socioeconomic factors.

Table S1: Fatality hazard ratios for hospitalized U.K. COVID-19 patients

Age	Hazard Ratio	Comorbidity	Hazard Ratio
20 to 49	1	Diabetes	1·1
50 to 59	2·7	Malignant Cancer	1·1
60 to 69	5·5	Chronic Cardiac Disease	1·2
70 to 79	9·8	Chronic Pulmonary Disease	1·2
80+	13·5	Chronic Kidney Disease	1·3
		Obesity	1·3
		Liver Disease	1·5

Source: Doherty et al. (2020), Figure 5.

Table S2: Comorbidity prevalence in New York City hospitalized COVID-19 patients vs. general population

Comorbidity	NYC Hospitalized COVID Patients	NYC Population (Ages 50+)	Difference
**Cancer**	5·6%	6·3%	-0·7%
**Cardiovascular Disease**			
Hypertension	53·1%	49·2%	3·9%
Coronary artery disease	10·4%	10·5%	-0·1%
Congestive heart failure	6·5%	6·9%	-0·4%
**Chronic Respiratory Disease**			
Asthma	8·4%	8·6%	-0·2%
Chronic obstructive pulmonary disease	5·0%	7·7%	-2·7%
Obstructive sleep apnea	2·7%	2·8%	-0·1%
**Immunosuppression**			
HIV	0·8%	2·7%	-2·0%
History of solid organ transplant	1·0%	NA	NA
**Kidney Disease**			
Chronic	4·7%	13·1%	-8·4%
End-Stage	3·3%	0·6%	2·6%
**Liver Disease**			
Cirrhosis	0·3%	0·9%	-0·6%
Hepatitis B	0·1%	0·5%	-0·3%
Hepatitis C	0·1%	0·1%	0·0%
**Metabolic Disease**			
Obesity (BMI>=30)	41·7%	26·9%	14·8%
Diabetes	31·7%	27·6%	4·1%
**Ever Smoked**	15·6%	43·8%	-28·2%

Note: The following sources were used to gauge the prevalence of comorbidities among NYC residents ages 50 years and above. *Asthma*: U.S. Center for Disease Control & Prevention (2018). *Cancer*: New York State Cancer Registry (2016). *Cardiovascular Diseases*: New York Department of Health (2020). *Diabetes*: New York State Comptroller (2015). *HIV*: New York City Department of Health (2018). *Kidney Disease*: IPRO End-Stage Renal Disease Network of New York (2014). *Liver Disease*: Moon et al. (2019) and Must et al. (1999). *Chronic Pulmonary Disease*: New York Department of Health (2019). *Obesity*: New York City Department of Health (2019).

Supplementary Appendix T: Metaregression Predictions for Manaus, Brazil

A recent seroprevalence study of Manaus, Brazil found prevalence of about 66% as of August 2020; moreover, that prevalence was roughly similar across age groups.[175] As of October 29, the Brazil Ministry of Health reported 2853 confirmed COVID-19 deaths in Manaus.[176] Assuming a uniform prevalence of 66%, the following table shows the age-specific predictions of our metaregression based on the age structure of the Manaus population. The predicted death toll of 3108 is well aligned with the actual number of confirmed COVID-19 deaths, and the predicted population IFR of 0·22% is well aligned with the observed population IFR of 0·2%.

Age Group	MedianAge	Population	Infections	Metaregression IFR (%)	Predicted Deaths
0-9 years	5	397,825	262,565	0·001	3
10-19 years	15	431,275	284,642	0·003	9
20-29 years	25	443,147	292,477	0·011	32
30-39 years	35	371,428	245,142	0·037	90
40-49 years	45	251,827	166,206	0·123	205
50-59 years	55	156,343	103,186	0·413	426
60-69 years	65	77,153	50,921	1·381	703
70+ years	75	53,764	35,484	4·622	1,640
All	28	2,182,763	1,440,623	0·216	3,108

Note: The total population of Manaus in 2020 is reported by the Brazil Ministry of Health. The age structure of the population is assumed to be identical to that of the 2010 census, downloaded from: *https://www.citypopulation.de/en/brazil/amazonas/manaus/130260305__manaus/* .

References for Supplementary Appendices

1. Moher D, Liberati A, Tetzlaff J, Altman DG. Preferred reporting items for systematic reviews and meta-analyses: the PRISMA statement. BMJ. 2009;339:b2535. doi:10.1136/bmj.b2535
2. University of Zurich Zika Open Access Project. Living Evidence on COVID-19. 2020. https://zika.ispm.unibe.ch/assets/data/pub/search_beta/. Accessed August 12 2020.
3. United Kingdom Parliament Office. Antibody Tests for COVID-19. 2020.
4. Byambasuren O, Dobler CC, Bell K, et al. Estimating the seroprevalence of SARS-CoV-2 infections: systematic review. 2020.
5. Ioannidis J. The infection fatality rate of COVID-19 inferred from seroprevalence data. 2020.
6. Meyerowitz-Katz G, Merone L. A systematic review and meta-analysis of published research data on COVID-19 infection-fatality rates. 2020.
7. Arora RK, Joseph A, Van Wyk J, et al. SeroTracker: a global SARS-CoV-2 seroprevalence dashboard. The Lancet Infectious Diseases. 2020. doi:10.1016/s1473-3099(20)30631-9
8. Lisboa Bastos M, Tavaziva G, Abidi SK, et al. Diagnostic accuracy of serological tests for covid-19: systematic review and meta-analysis. BMJ. 2020;370:m2516. doi:10.1136/bmj.m2516
9. Rogan WJ, Gladen B. ESTIMATING PREVALENCE FROM THE RESULTS OF A SCREENING TEST. American Journal of Epidemiology. 1978;107(1):71-6. doi:10.1093/oxfordjournals.aje.a112510
10. Havers FP, Reed C, Lim T, et al. Seroprevalence of Antibodies to SARS-CoV-2 in 10 Sites in the United States, March 23-May 12, 2020. JAMA Intern Med. 2020. doi:10.1001/jamainternmed.2020.4130
11. Rosenberg ES, Tesoriero JM, Rosenthal EM, et al. Cumulative incidence and diagnosis of SARS-CoV-2 infection in New York. annals of epidemiology. 2020. doi:10.1016/J.ANNEPIDEM.2020.06.004
12. Stringhini S, Wisniak A, Piumatti G, et al. Seroprevalence of anti-SARS-CoV-2 IgG antibodies in Geneva, Switzerland (SEROCoV-POP): a population-based study. The Lancet. 2020;396(10247):313-9. doi:10.1016/s0140-6736(20)31304-0
13. United Kingdom Office for National Statistics. Coronavirus (COVID-19) infection survey: characteristics of people testing positive for COVID-19 in England, August 2020. 2020.
14. University of Utah Health. Utah COVID-19 update: preliminary results from the Utah HERO project. 25:10 ed2020.
15. Ward H, Atchison CJ, Whitaker M, et al. Antibody prevalence for SARS-CoV-2 in England following first peak of the pandemic: REACT2 study in 100,000 adults. medRxiv. 2020:2020.08.12.20173690. doi:10.1101/2020.08.12.20173690
16. Italy National Institute of Statistics. Primi risultati dell'indagine di sieroprevalenza sul SARS-CoV-2. 2020.
17. Merkely B, Szabo AJ, Kosztin A, et al. Novel coronavirus epidemic in the Hungarian population, a cross-sectional nationwide survey to support the exit policy in Hungary. Geroscience. 2020;42(4):1063-74. doi:10.1007/s11357-020-00226-9
18. Portugal National Institute of Health. Relatório de Apresentação dos Resultados Preliminares do Primeiro Inquérito Serológico Nacional COVID-19. 2020.
19. Menachemi N, Yiannoutsos CT, Dixon BE, et al. Population Point Prevalence of SARS-CoV-2 Infection Based on a Statewide Random Sample — Indiana, April 25–29, 2020. morbidity and mortality weekly report. 2020;69(29):960-4. doi:10.15585/MMWR.MM6929E1
20. Pollán M, Pérez-Gómez B, Pastor-Barriuso R, et al. Prevalence of SARS-CoV-2 in Spain (ENE-COVID): a nationwide, population-based seroepidemiological study. The Lancet. 2020. doi:10.1016/s0140-6736(20)31483-5
21. U.S. Food and Drug Administration. EUA authorized serology test performance. 2020.

22. Larremore DB, Fosdick BK, Bubar KM, et al. Estimating SARS-CoV-2 seroprevalence and epidemiological parameters with uncertainty from serological surveys. 2020. doi:10.1101/2020.04.15.20067066
23. Manski CF, Molinari F. Estimating the COVID-19 infection rate: Anatomy of an inference problem. J Econom. 2020. doi:10.1016/j.jeconom.2020.04.041
24. Herzog S, Bie JD, Abrams S, et al. Seroprevalence of IgG antibodies against SARS coronavirus 2 in Belgium: a prospective cross-sectional study of residual samples. 2020.
25. Carrat F, de Lamballerie X, Rahib D, et al. Seroprevalence of SARS-CoV-2 among adults in three regions of France following the lockdown and associated risk factors: a multicohort study. medRxiv. 2020:2020.09.16.20195693. doi:10.1101/2020.09.16.20195693
26. Ireland Health Service Executive. Preliminary report of the results of the Study to Investigate COVID-19 Infection in People Living in Ireland (SCOPI): a national seroprevalence study, June-July 2020. 2020.
27. Our World in Data. Coronavirus (COVID-19) testing: tests per confirmed case. 2020. https://ourworldindata.org/coronavirus-testing#tests-per-confirmed-case. Accessed August 18.
28. Gudbjartsson DF, Norddahl GL, Melsted P, et al. Humoral Immune Response to SARS-CoV-2 in Iceland. New England Journal of Medicine. 2020. doi:10.1056/NEJMoa2026116
29. Korea Center for Disease Control. Update on COVID-19 in Korea as of 14 September. 2020.
30. Noh JY, Seo YB, Yoon JG, et al. Seroprevalence of Anti-SARS-CoV-2 Antibodies among Outpatients in Southwestern Seoul, Korea. Journal of Korean medical science. 2020;35(33):e311-e. doi:10.3346/jkms.2020.35.e311
31. Iceland Directorate of Health. COVID-19 in Iceland - Statistics 28 Feb to 14 June 2020. 2020.
32. Gudbjartsson DF, Helgason A, Jonsson H, et al. Spread of SARS-CoV-2 in the Icelandic Population. N Engl J Med. 2020;382(24):2302-15. doi:10.1056/NEJMoa2006100
33. Molenberghs G, Faes C, Aerts J, et al. Belgian Covid-19 Mortality, Excess Deaths, Number of Deaths per Million, and Infection Fatality Rates (8 March - 9 May 2020). 2020.
34. European Centre for Disease Prevention and Control. Clinical characteristics of COVID-19. 2020.
35. Fouillet A. Surveillance de la mortalité au cours de l'épidémie de COVID-19 du 2 mars au 31 mai 2020 en France. In: France Sp, editor.2020. p. 83.
36. France Public Health. Special COVID-19 point épidémio régional: Grand Est, 04 juin 2020. 2020.
37. France Public Health. Spécial COVID-19 point épidémio régional: Ile-de-France, 28 mai 2020. 2020.
38. France Public Health. Spécial COVID-19 point épidémio régional: Nouvell-Aquitaine, 28 mai 2020. 2020.
39. Perez-Saez J, Lauer SA, Kaiser L, et al. Serology-informed estimates of SARS-CoV-2 infection fatality risk in Geneva, Switzerland. The Lancet Infectious Diseases. doi:10.1016/S1473-3099(20)30584-3
40. Ireland Health Service Executive. Epidemiology of COVID-19 in Ireland: Report prepared by HPSC on 04/08/2020 for National Public Health Emergency Team. 2020.
41. Pastor-Barriuso R, Perez-Gomez B, Hernan MA, et al. SARS-CoV-2 infection fatality risk in a nationwide seroepidemiological study. medRxiv. 2020:2020.08.06.20169722. doi:10.1101/2020.08.06.20169722
42. Abellán A, Aceituno P, Fernández I, Ramiro D, Pujol R. Una estimación de la población que vive en residencias de mayores. Envejecimiento en red. 2020.
43. EuroStat. Weekly death statistics. 2020.
44. France Institute for Demographic Studies (INED). Demographics of COVID-19 deaths. 2020.
45. Ontario Public Health. COVID-19 case data as of July 15. 2020.
46. Sweden Public Health Authority. COVID-19 Report for Week 24 - COVID-19 veckorapport vecka 24. 2020.
47. Georgia Department of Public Health. COVID-19 Daily Status Report 2020.

48. Connecticut Department of Health & Human Services. COVID-19 cases and deaths by age group. 2020.
49. Indiana State Department of Public Health. Indiana COVID-19 data report – demographic distributions. 2020.
50. Louisiana Department of Health. Louisiana coronavirus (COVID-19) information – cases/deaths by age group. 2020.
51. Florida Department of Health. Florida COVID-19 case line data. 2020.
52. Minnesota Department of Health. COVID-19 newly reported deaths detail - age group data table including age group of deaths. 2020.
53. Missouri Department of Health & Senior Services. Missouri COVID-19 dashboard. 2020.
54. New York Department of Health. COVID-19 Tracker – Fatalities by Age Group. 2020.
55. Pennsylvania Department of Health. COVID-19 death data for Pennsylvania. 2020.
56. Pennsylvania Department of Health. Weekly report for deaths attributed to COVID-19. 2020.
57. Utah Department of Health. COVID-19 surveillance. 2020.
58. California Department of Public Health. Data dashboards - COVID-19 in the state. 2020.
59. Washington Department of Health. Novel coronavirus outbreak (COVID-19) cases and deaths by week of illness onset, county, and age. 2020.
60. Australia Department of Health. Coronavirus (COVID-19) current situation and case numbers. 2020.
61. Korea Center for Disease Control. Weekly Report on the COVID-19 Situation in the Republic of Korea. 2020.
62. Lithuania Central Registry. Koronaviruso (COVID-19) Lietuvoje statistika. 2020.
63. New Zealand Ministry of Health. COVID-19 Current Cases. 2020.
64. Harbord RM, Higgins JPT. Meta-regression in Stata. Stata Journal. 2008;8(4):493-519.
65. Higgins JPT, Thompson SG, Spiegelhalter DJ. A re-evaluation of random-effects meta-analysis. J R Stat Soc Ser A Stat Soc. 2009;172(1):137-59. doi:10.1111/j.1467-985X.2008.00552.x
66. Bonanad C, Garcia-Blas S, Tarazona-Santabalbina F, et al. The Effect of Age on Mortality in Patients With COVID-19: A Meta-Analysis With 611,583 Subjects. J Am Med Dir Assoc. 2020;21(7):915-8. doi:10.1016/j.jamda.2020.05.045
67. Docherty AB, Harrison EM, Green CA, et al. Features of 20 133 UK patients in hospital with covid-19 using the ISARIC WHO Clinical Characterisation Protocol: prospective observational cohort study. BMJ. 2020;369:m1985. doi:10.1136/bmj.m1985
68. CBC News. Serology study gives officials first look at spread of COVID-19 through Alberta's population. 2020 July 30.
69. Giustizia News. Coronavirus, l'incubo senza fine di Ariano Irpino: 60 positivi al tampone dopo i test sierologici. May 28.
70. Hicks S, Pohl K, Neeman T, et al. A dual antigen ELISA allows the assessment of SARS-CoV-2 antibody seroprevalence in a low transmission setting. medRxiv. 2020:2020.09.09.20191031. doi:10.1101/2020.09.09.20191031
71. Austria Statistik. COVID-19 prevalence study: a maximum of 0.15% of the population in Austria infected with SARS-CoV-2. 2020.
72. Robert Koch Institut. Corona-Monitoring lokal - erste eckdaten fur Bad Feilnbach. 2020.
73. Feehan AK, Velasco C, Fort D, et al. Racial and workplace disparities in seroprevalence of SARS-CoV-2 in Baton Rouge, Louisiana, July 15-31, 2020. medRxiv. 2020:2020.08.26.20180968. doi:10.1101/2020.08.26.20180968
74. McLaughlin CC, Doll MK, Morrison KT, et al. High Community SARS-CoV-2 Antibody Seroprevalence in a Ski Resort Community, Blaine County, Idaho, US. Preliminary Results. medRxiv. 2020. doi:10.1101/2020.07.19.20157198

75. Appa A, Takahashi S, Rodriguez-Barraquer I, et al. Universal PCR and antibody testing demonstrate little to no transmission of SARS-CoV-2 in a rural community. medRxiv. 2020:2020.08.15.20175786. doi:10.1101/2020.08.15.20175786
76. Toenshoff B, Muller B, Elling R, et al. Prevalence of SARS-CoV-2 Infection in Children and Their Parents in Southwest Germany. 2020. doi:http://dx.doi.org/10.2139/ssrn.3668418
77. Skowronski DM, Sekirov I, Sabaiduc S, et al. Low SARS-CoV-2 sero-prevalence based on anonymized residual sero-survey before and after first wave measures in British Columbia, Canada, March-May 2020. 2020.
78. van den Broek-Altenburg E, Atherly A, Diehl S, et al. Risk Factors for COVID-19: Community Exposure and Mask-Wearing. 2020. doi:https://dx.doi.org/10.2139/ssrn.3676570
79. Micolitti A. Caldari Ortona: 12% cittadini sottoposti a test sierologici positivi con anticorpi al Covid 19. Rete8. 2020 June 3.
80. Naranbhai V, Chang CC, Beltran WFG, et al. High seroprevalence of anti-SARS-CoV-2 antibodies in Chelsea, Massachusetts. The Journal of Infectious Diseases. 2020. doi:10.1093/infdis/jiaa579
81. Mahajan S, Srinivasan R, Redlich CA, et al. Seroprevalence of SARS-CoV-2-Specific IgG Antibodies Among Adults Living in Connecticut Between March 1 and June 1, 2020: Post-Infection Prevalence (PIP) Study. medRxiv. 2020:2020.08.04.20168203. doi:10.1101/2020.08.04.20168203
82. Czech Ministry of Health. Collective Immunity Study SARS-CoV-2: Czech Prevalence. 2020.
83. Denmark State Blood Institute. Notat: Nye foreløbige resultater fra den repræsentative seroprævalensundersøgelse af COVID-19. 2020.
84. Petersen MS, Strøm M, Christiansen DH, et al. Seroprevalence of SARS-CoV-2–Specific Antibodies, Faroe Islands. emerging infectious diseases. 2020;26(11). doi:10.3201/EID2611.202736
85. Finland National Institute for Health and Welfare. Weekly report of THL serological population study of the coronavirus epidemic. 2020.
86. Streeck H, Schulte B, Kuemmerer B, et al. Infection fatality rate of SARS-CoV-2 infection in a German community with a super-spreading event. 2020.
87. Bogogiannidou Z, Vontas A, Dadouli K, et al. Repeated leftover serosurvey of SARS-CoV-2 IgG antibodies, Greece, March and April 2020. Eurosurveillance. 2020;25(31):2001369. doi:doi:https://doi.org/10.2807/1560-7917.ES.2020.25.31.2001369
88. Hibino S, Hayashida K, Ahn AC, Hayashida Y. Dynamic Change of COVID-19 Seroprevalence among Asymptomatic Population in Tokyo during the Second Wave. medRxiv. 2020:2020.09.21.20198796. doi:10.1101/2020.09.21.20198796
89. Northeast Texas Public Health Department. Hideaway COVID-19 antibody testing program. 2020.
90. Knabl L, Mitra T, Kimpel J, et al. High SARS-CoV-2 Seroprevalence in Children and Adults in the Austrian Ski Resort Ischgl. medRxiv. 2020:2020.08.20.20178533. doi:10.1101/2020.08.20.20178533
91. Times of Israel Staff. Coronavirus : Israël est encore loin de l'immunité de groupe. Times of Israel. 2020 July 23.
92. Nishiura H, Kobayashi T, Yang Y, et al. The Rate of Underascertainment of Novel Coronavirus (2019-nCoV) Infection: Estimation Using Japanese Passengers Data on Evacuation Flights. J Clin Med. 2020;9(2). doi:10.3390/jcm9020419
93. Jersey Health & Community Services. Prevalence of Antibodies – Community Survey Round 2. 2020.
94. Figueiredo-Campos P, Blankenhaus B, Mota C, et al. Seroprevalence of anti-SARS-CoV-2 antibodies in COVID-19 patients and healthy volunteers. medRxiv. 2020:2020.08.30.20184309. doi:10.1101/2020.08.30.20184309

95. University of Louisville School of Medicine. Phase II results of Co-Immunity Project show higher-than-expected rates of exposure to novel coronavirus in Jefferson County. 2020.
96. University of Miami. SPARK-C: understanding the burden of COVID-19 in Miami-Dade County through rapid serological testing of a representative random sample. 2020.
97. Stadlbauer D, Tan J, Jiang K, et al. Seroconversion of a city: Longitudinal monitoring of SARS-CoV-2 seroprevalence in New York City. medRxiv. 2020:2020.06.28.20142190. doi:10.1101/2020.06.28.20142190
98. Weis S, Scherag A, Baier M, et al. Seroprevalence of SARS-CoV-2 antibodies in an entirely PCR-sampled and quarantined community after a COVID-19 outbreak - the CoNAN study. 2020.
99. Feehan AK, Fort D, Garcia-Diaz J, et al. Seroprevalence of SARS-CoV-2 and Infection Fatality Ratio, Orleans and Jefferson Parishes, Louisiana, USA, May 2020. Emerg Infect Dis. 2020;26(11). doi:10.3201/eid2611.203029
100. Yang W, Kandula S, Huynh M, et al. Estimating the infection fatality risk of COVID-19 in New York City during the spring 2020 pandemic wave. medRxiv. 2020:2020.06.27.20141689. doi:10.1101/2020.06.27.20141689
101. Norrbotten Region. Forekomst av antikroppar mot covid-19 - Norrbottens befolkning maj 2020. 2020.
102. Health NIoP. Seroprevalence of SARS-CoV-2 in the Norwegian population measured in residual sera collected in April/May 2020 and August 2019. 2020.
103. Dimeglio C, Loubes J-M, Miedougé M, Herin F, Soulat J-M, Izopet J. The real seroprevalence of SARS-CoV-2 in France and its consequences for virus dynamics. Research Square; 2020.
104. Oklahoma State Department of Health. Weekly Epidemiology and Surveillance Report. 2020.
105. Norway Public Health Institute. Truleg berre ein liten andel av befolkninga som har vore smitta av koronavirus. 2020.
106. Ripperger TJ, Uhrlaub JL, Watanabe M, et al. Detection, prevalence, and duration of humoral responses to SARS-CoV-2 under conditions of limited population exposure. medRxiv. 2020:2020.08.14.20174490. doi:10.1101/2020.08.14.20174490
107. Rhode Island Department of Health. COVID-19 serology testing brief. 2020.
108. Riverside County Joint Information Center. Antibody study shows coronavirus spread wider in Riverside County. 2020.
109. Chamie G, Marquez C, Crawford E, et al. SARS-CoV-2 Community Transmission During Shelter-in-Place in San Francisco. 2020.
110. San Miguel County Department of Health & Environment. IgG Antibody Tests: Statistics and Demographics. 2020.
111. Slovenia Government Communication Office. First study carried out on herd immunity of the population in the whole territory of Slovenia. 2020.
112. Wells PM, Doores KM, Couvreur S, et al. Estimates of the rate of infection and asymptomatic COVID-19 disease in a population sample from SE England. 2020.
113. Sweden Public Health Authority. The Infection Fatality Rate of COVID-19 in Stockholm – Technical Report. 2020.
114. Lundkvist Å, Hanson S, Olsen B. Pronounced difference in Covid-19 antibody prevalence indicates cluster transmission in Stockholm, Sweden. Infection Ecology & Epidemiology. 2020;10(1):1806505. doi:10.1080/20008686.2020.1806505
115. Stockholm Region. Lägesrapport om arbetet med det nya coronaviruset. 2020.
116. Japan Ministry of Health Labour and Welfare. Updates on COVID-19 in Japan. 2020.
117. U.S. Center for Disease Control and Prevention. COVIDView weekly report - U.S. virologic surveillance by public health laboratories. 2020.
118. Nawa N, Kuramochi J, Sonoda S, et al. Seroprevalence of SARS-CoV-2 IgG Antibodies in Utsunomiya City, Greater Tokyo, after first pandemic in 2020 (U-CORONA): a household- and population-based study. 2020.

119. Virginia Office of the Governor. Governor Northam announces launch of pediatric coronavirus serology study; interim adult serology study findings show an estimated 2.4% of adult Virginians have COVID-19 antibodies. 2020.
120. Lavezzo E, Franchin E, Ciavarella C, et al. Suppression of COVID-19 outbreak in the municipality of Vo, Italy. 2020.
121. Washoe County Health District. Seroprevalence of SARS-CoV-2 Specific Antibodies Among Adults in Washoe County, Nevada on June 9-10, 2020. 2020.
122. Wake Forest Baptist Health. COVID-19 community research partnership study results and data. 2020.
123. Fenwick C, Croxatto A, Coste AT, et al. Changes in SARS-CoV-2 Antibody Responses Impact the Estimates of Infections in Population-Based Seroprevalence Studies. medRxiv. 2020:2020.07.14.20153536. doi:10.1101/2020.07.14.20153536
124. Sood N, Simon P, Ebner P, et al. Seroprevalence of SARS-CoV-2-Specific Antibodies Among Adults in Los Angeles County, California, on April 10-11, 2020. JAMA. 2020. doi:10.1001/jama.2020.8279
125. Snoeck CJ, Vaillant M, Abdelrahman T, et al. Prevalence of SARS-CoV-2 infection in the Luxembourgish population: the CON-VINCE study. 2020.
126. Bryan A, Pepper G, Wener MH, et al. Performance Characteristics of the Abbott Architect SARS-CoV-2 IgG Assay and Seroprevalence in Boise, Idaho. J Clin Microbiol. 2020;58(8). doi:10.1128/JCM.00941-20
127. Bendavid E, Mulaney B, Sood N, et al. COVID-19 Antibody Seroprevalence in Santa Clara County, California. 2020.
128. Kekatos M. Stanford University is investigating its own researchers over claims their antibody study was politically motivated and 'tipped the scale' to make COVID-19 seem less lethal. Daily Mail. 2020 May 26.
129. Kraehling V, Kern M, Halwe S, et al. Epidemiological study to detect active SARS-CoV-2 infections and seropositive persons in a selected cohort of employees in the Frankfurt am Main metropolitan area. 2020.
130. Reifer J, Hayum N, Heszkel B, Klagsbald I, Streva VA. SARS-CoV-2 IgG antibody responses in New York City. diagnostic microbiology and infectious disease. 2020. doi:10.1016/J.DIAGMICROBIO.2020.115128
131. Doi A, Iwata K, Kuroda H, et al. Estimation of seroprevalence of novel coronavirus disease (COVID-19) using preserved serum at an outpatient setting in Kobe, Japan: A cross-sectional study. 2020.
132. Takita M, Matsumura T, Yamamoto K, et al. Geographical Profiles of COVID-19 Outbreak in Tokyo: An Analysis of the Primary Care Clinic-Based Point-of-Care Antibody Testing. J Prim Care Community Health. 2020;11:2150132720942695. doi:10.1177/2150132720942695
133. Takita M, Matsumura T, Yamamoto K, et al. Challenges of community point-of-care antibody testing for COVID-19 herd-immunity in Japan. QJM. 2020. doi:10.1093/qjmed/hcaa182
134. Anand S, Montez-Rath M, Han J, et al. Prevalence of SARS-CoV-2 antibodies in a large nationwide sample of patients on dialysis in the USA: a cross-sectional study. The Lancet. doi:10.1016/S0140-6736(20)32009-2
135. Emmenegger M, Cecco ED, Lamparter D, et al. Population-wide evolution of SARS-CoV-2 immunity tracked by a ternary immunoassay. 2020.
136. Fiore J, Centra M, de Carlo A, et al. Far away from herd immunity to SARS-CoV-2: results from a survey in healthy blood donors in southeastern Italy. 2020. doi:10.1101/2020.06.17.20133678
137. Canadian Blood Services. COVID-19 seroprevalence report-August 19, 2020. 2020.
138. Erikstrup C, Hother CE, Pedersen OBV, et al. Estimation of SARS-CoV-2 infection fatality rate by real-time antibody screening of blood donors. clinical infectious diseases. 2020. doi:10.1093/CID/CIAA849

139. Reuters Health News. Denmark to send back inaccurate antibody tests from China's Livzon. 2020 May 20.
140. Public Health England. Sero-Surveillance of COVID-19: Week 22. 2020.
141. Fischer B, Knabbe C, Vollmer T. SARS-CoV-2 IgG seroprevalence in blood donors located in three different federal states, Germany, March to June 2020. Euro Surveill. 2020;25(28). doi:10.2807/1560-7917.ES.2020.25.28.2001285
142. Percivalle E, Cambie G, Cassaniti I, et al. Prevalence of SARS-CoV-2 specific neutralising antibodies in blood donors from the Lodi Red Zone in Lombardy, Italy, as at 06 April 2020. Euro Surveill. 2020;25(24). doi:10.2807/1560-7917.ES.2020.25.24.2001031
143. Valenti L, Bergna A, Pelusi S, et al. SARS-CoV-2 seroprevalence trends in healthy blood donors during the COVID-19 Milan outbreak. 2020.
144. Slot E, Hogema BM, Reusken CBEM, et al. Herd immunity is not a realistic exit strategy during a COVID-19 outbreak. 2020. doi:10.21203/rs.3.rs-25862/v1
145. Nesbitt DJ, Jin D, Hogan JW, et al. Low Seroprevalence of SARS-CoV-2 in Rhode Island Blood Donors Determined using Multiple Serological Assay Formats. 2020.
146. Thompson CP, Grayson N, Paton R, et al. Detection of neutralising antibodies to SARS coronavirus 2 to determine population exposure in Scottish blood donors between March and May 2020. medRxiv. 2020:2020.04.13.20060467. doi:10.1101/2020.04.13.20060467
147. Ng D, Goldgof G, Shy B, et al. SARS-CoV-2 seroprevalence and neutralizing activity in donor and patient blood from the San Francisco Bay Area. medRxiv. 2020. doi:10.1101/2020.05.19.20107482
148. Vassallo RR, Bravo MD, Dumont LJ, Hazegh K, Kamel H. Seroprevalence of Antibodies to SARS-CoV-2 in US Blood Donors. medRxiv. 2020:2020.09.17.20195131. doi:10.1101/2020.09.17.20195131
149. Dodd RY, Xu M, Stramer SL. Change in Donor Characteristics and Antibodies to SARS-CoV-2 in Donated Blood in the US, June-August 2020. JAMA. 2020. doi:10.1001/jama.2020.18598
150. Fontanet A, Tondeur L, Madec Y, et al. Cluster of COVID-19 in northern France: A retrospective closed cohort study. medRxiv. 2020:2020.04.18.20071134. doi:10.1101/2020.04.18.20071134
151. Armann JP, Unrath M, Kirsten C, Lueck C, Dalpke A, Berner R. Anti-SARS-CoV-2 IgG antibodies in adolescent students and their teachers in Saxony, Germany (SchoolCoviDD19): very low seropraevalence and transmission rates. 2020. doi:10.1101/2020.07.16.20155143
152. Ulyte A, Radtke T, Abela IA, et al. Variation in SARS-CoV-2 seroprevalence in school-children across districts, schools and classes. medRxiv. 2020:2020.09.18.20191254. doi:10.1101/2020.09.18.20191254
153. Rigatti SJ, Stout R. SARS-CoV-2 Antibody Prevalence and Association with Routine Laboratory Values in a Life Insurance Applicant Population. medRxiv. 2020:2020.09.09.20191296. doi:10.1101/2020.09.09.20191296
154. Poletti P, Tirani M, Cereda D, et al. Age-specific SARS-CoV-2 infection fatality ratio and associated risk factors, Italy, February to April 2020. Euro surveillance : bulletin Europeen sur les maladies transmissibles = European communicable disease bulletin. 2020;25(31):2001383. doi:10.2807/1560-7917.ES.2020.25.31.2001383
155. Biggs HM. Estimated Community Seroprevalence of SARS-CoV-2 Antibodies — Two Georgia Counties, April 28–May 3, 2020. morbidity and mortality weekly report. 2020;69(29):965-70. doi:10.15585/MMWR.MM6929E2
156. Sutton M, Cieslak P, Linder M. Seroprevalence Estimates of SARS-CoV-2 Infection in Convenience Sample — Oregon, May 11–June 15, 2020. Morbidity and Mortality Weekly Report.69:1100-1. doi:http://dx.doi.org/10.15585/mmwr.mm6932a4
157. Waterfield T, Watson C, Moore R, et al. Seroprevalence of SARS-CoV-2 antibodies in children - A prospective multicentre cohort study. medRxiv. 2020:2020.08.31.20183095. doi:10.1101/2020.08.31.20183095

158. Netherlands National Institute for Public Health and the Environment. Children and COVID-19. 2020.
159. Ontario Public Health. COVID-19 seroprevalence in Ontario: March 27, 2020 to June 30, 2020. 2020.
160. University of Utah Health. Utah HERO project announces phase one findings. 2020.
161. University of Utah Health. COVID-19 Update: Utah HERO project results. 25:10 to 28:45 ed2020.
162. France Public Health. COVID-19 : point épidémiologique du 9 juillet 2020. 2020.
163. Pagani G, Conti F, Giacomelli A, et al. Seroprevalence of SARS-CoV-2 significantly varies with age: Preliminary results from a mass population screening. Journal of Infection. doi:10.1016/j.jinf.2020.09.021
164. Mizumoto K, Kagaya K, Zarebski A, Chowell G. Estimating the asymptomatic proportion of coronavirus disease 2019 (COVID-19) cases on board the Diamond Princess cruise ship, Yokohama, Japan, 2020. eurosurveillance. 2020;25(10). doi:10.2807/1560-7917.ES.2020.25.10.2000180
165. United Kingdom BioBank. UK Biobank SARS-CoV-2 Serology Study Weekly Report - 21 July 2020. 2020.
166. World Health Organization. Coronavirus disease (COVID-19): Similarities and differences with influenza. 2020.
167. U.S. Center for Disease Control and Prevention. Disease Burden of Influenza. 2020.
168. Furuya-Kanamori L, Cox M, Milinovich GJ, Magalhaes RJS, Mackay IM, Yakob L. Heterogeneous and Dynamic Prevalence of Asymptomatic Influenza Virus Infections. Emerging infectious diseases. 2016;22(6):1052-6. doi:10.3201/eid2206.151080
169. EuroMoMo. Excess Mortality in Europe. 2020.
170. Verity R, Okell LC, Dorigatti I, et al. Estimates of the severity of coronavirus disease 2019: a model-based analysis. lancet infectious diseases. 2020;20(6):669-77. doi:10.1016/S1473-3099(20)30243-7
171. Ferguson N, Laydon D, Nedjati-Gilani G, et al. Report 9: Impact of non-pharmaceutical interventions (NPIs) to reduce COVID-19 mortality and healthcare demand2020.
172. Williamson EJ, Walker AJ, Bhaskaran K, et al. Factors associated with COVID-19-related death using OpenSAFELY. Nature. 2020. doi:10.1038/s41586-020-2521-4
173. Richardson S, Hirsch JS, Narasimhan M, et al. Presenting Characteristics, Comorbidities, and Outcomes Among 5700 Patients Hospitalized With COVID-19 in the New York City Area. JAMA. 2020;323(20):2052-9. doi:10.1001/jama.2020.6775
174. Azar K, Shen Z, Romanelli R, et al. Disparities in outcomes among COVID-19 patients in a large health care system in California. Health Affairs. 2020;39. doi:https://www.healthaffairs.org/doi/10.1377/hlthaff.2020.00598
175. Buss LF, Prete CA, Abrahim CMM, et al. COVID-19 herd immunity in the Brazilian Amazon. medRxiv. 2020:2020.09.16.20194787. doi:10.1101/2020.09.16.20194787
176. Brazil Ministry of Health. COVID-19 No Brasil, Obitos Acumulados - Manaus. 2020.
